# Supplementary figures and images for: Atypical integrative element with strand-biased circularization activity assists interspecies antimicrobial resistance gene transfer from Vibrio alfacsensis
Source: PLoS One. 2022 Aug 2;17(8):e0271627. doi: 10.1371/journal.pone.0271627 (PMC9345347; doi:10.1371/journal.pone.0271627)

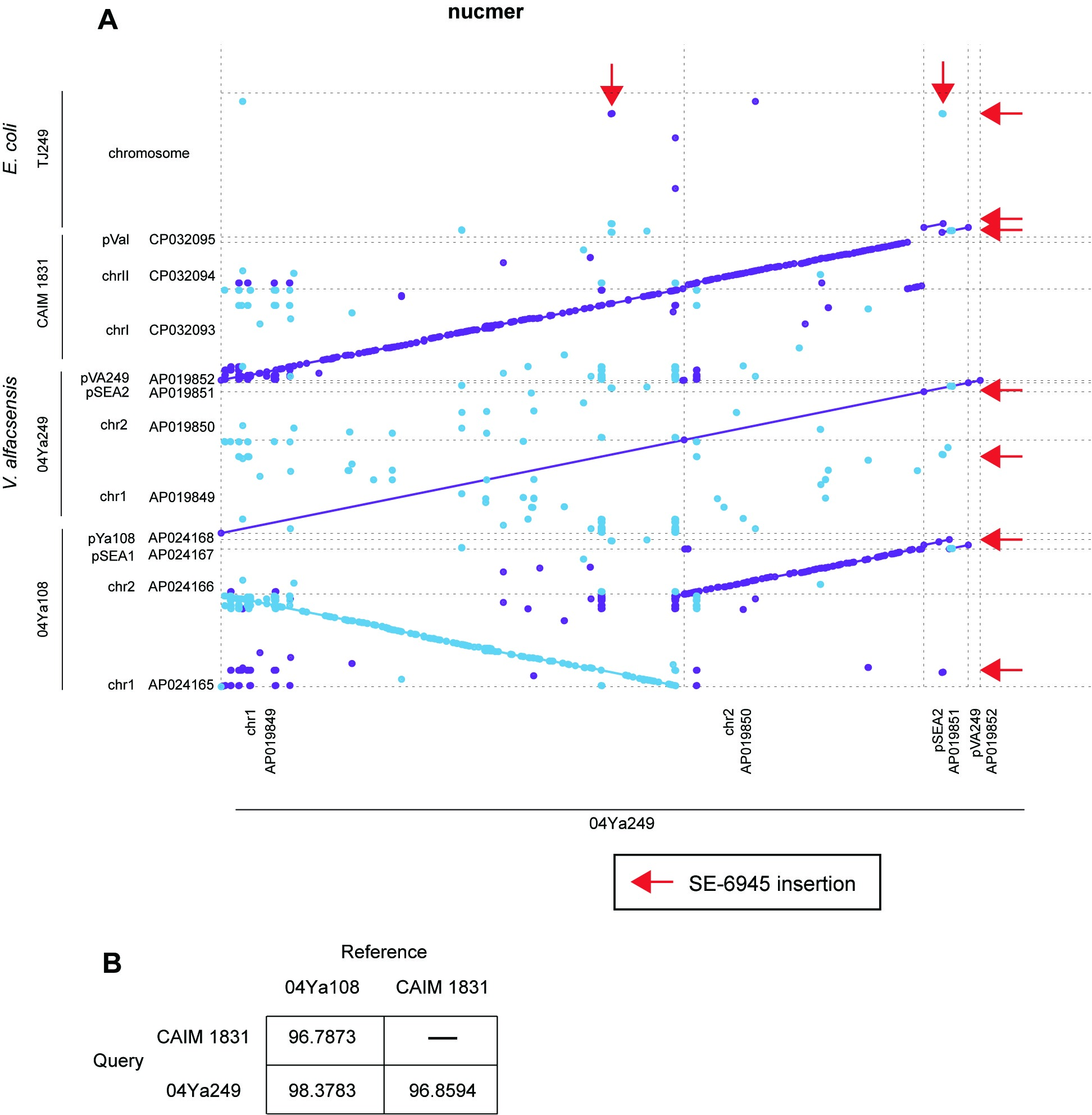

Supplement: S1 Fig — (A) Structure comparison was performed using nucmer in MUMmer3 [74]. Purple dots indicate a match on the Watson strand (5′ to 3′ on the top strand in GenBank file), and light blue indicates a match on the Crick strand (5′ to 3′ on the bottom strand). (B) Average nucleotide identity (ANI) between two strains as determined by fastANI [77]. The commands used were as follows: (A) $nucmer -minmatch 60‥/‥/data/04Ya249_submission.fas‥/‥/data/reference.fas$mummerplot -x "[0,6000000]" -y "[0,22000000]" -postscript -p test out.delta; (B) $fastANI -q‥/‥/data/04Ya249_submission.fas -r‥/‥/data/CAIM1831_Refseq.fas -o 04Ya249vsCAIM1831.txt $fastANI -q‥/‥/data/04Ya249_submission.fas -r‥/‥/data/04Ya108_submission.fas -o 04Ya249vs04Ya108.txt$fastANI -q‥/‥/data/04Ya249_submission.fas -r‥/‥/data/04Ya108_submission.fas -o 04Ya249vs04Ya108.txt. (TIF) [file pone.0271627.s001.tif]

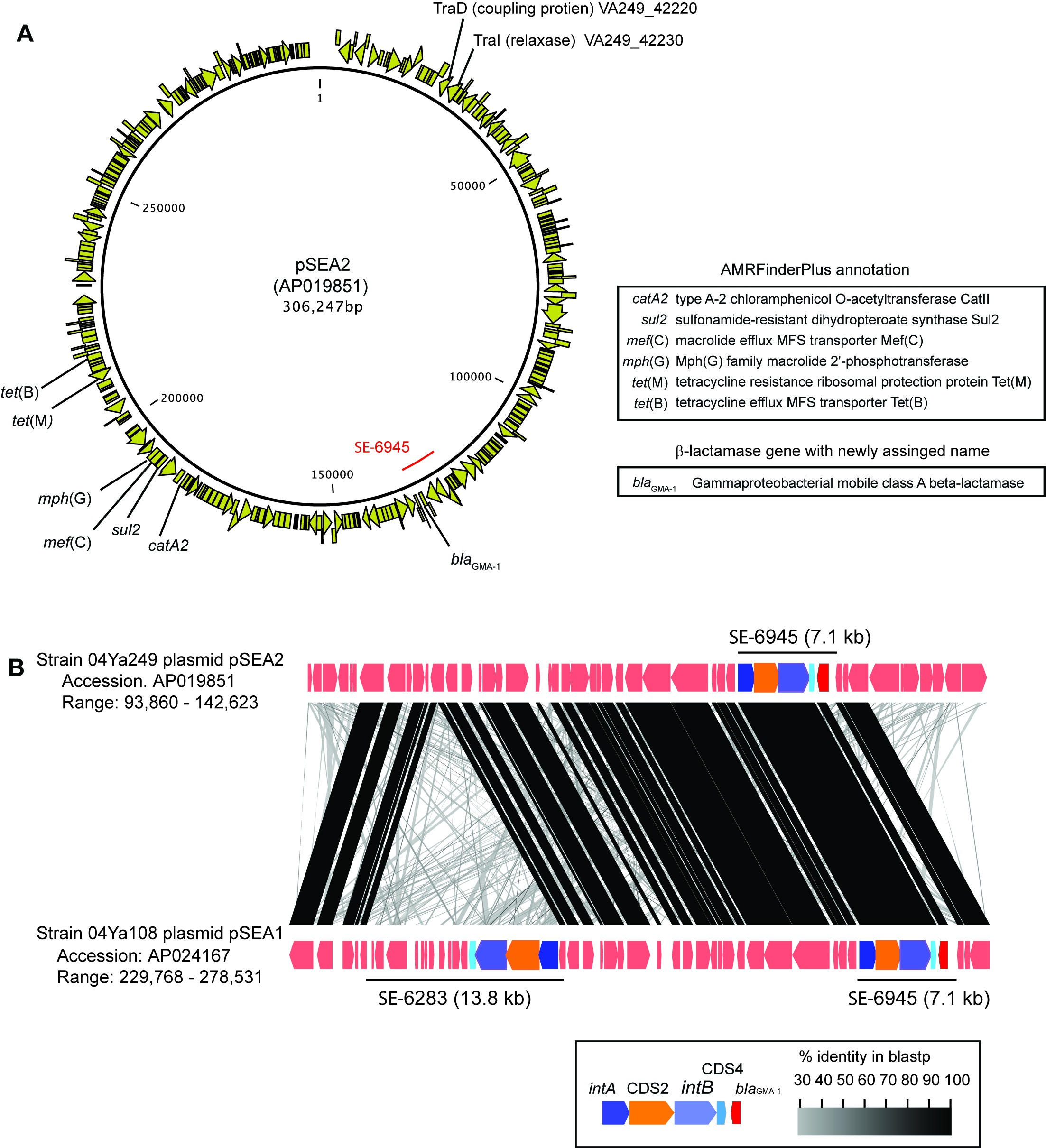

Supplement: S2 Fig — (A) Locations of antimicrobial resistance (AMR) genes and SE-6945 in pSEA2. AMR genes were inferred using AMRFinderPlus [76]. Genes were visualized using CLC Sequence Viewer (Qiagen, Hilden Germany). (B) Location of SEs in pSEA1 and pSEA2. Four SE core genes are indicated by four distinct colors. Red pentagons are the β-lactamase gene. (TIF) [file pone.0271627.s002.tif]

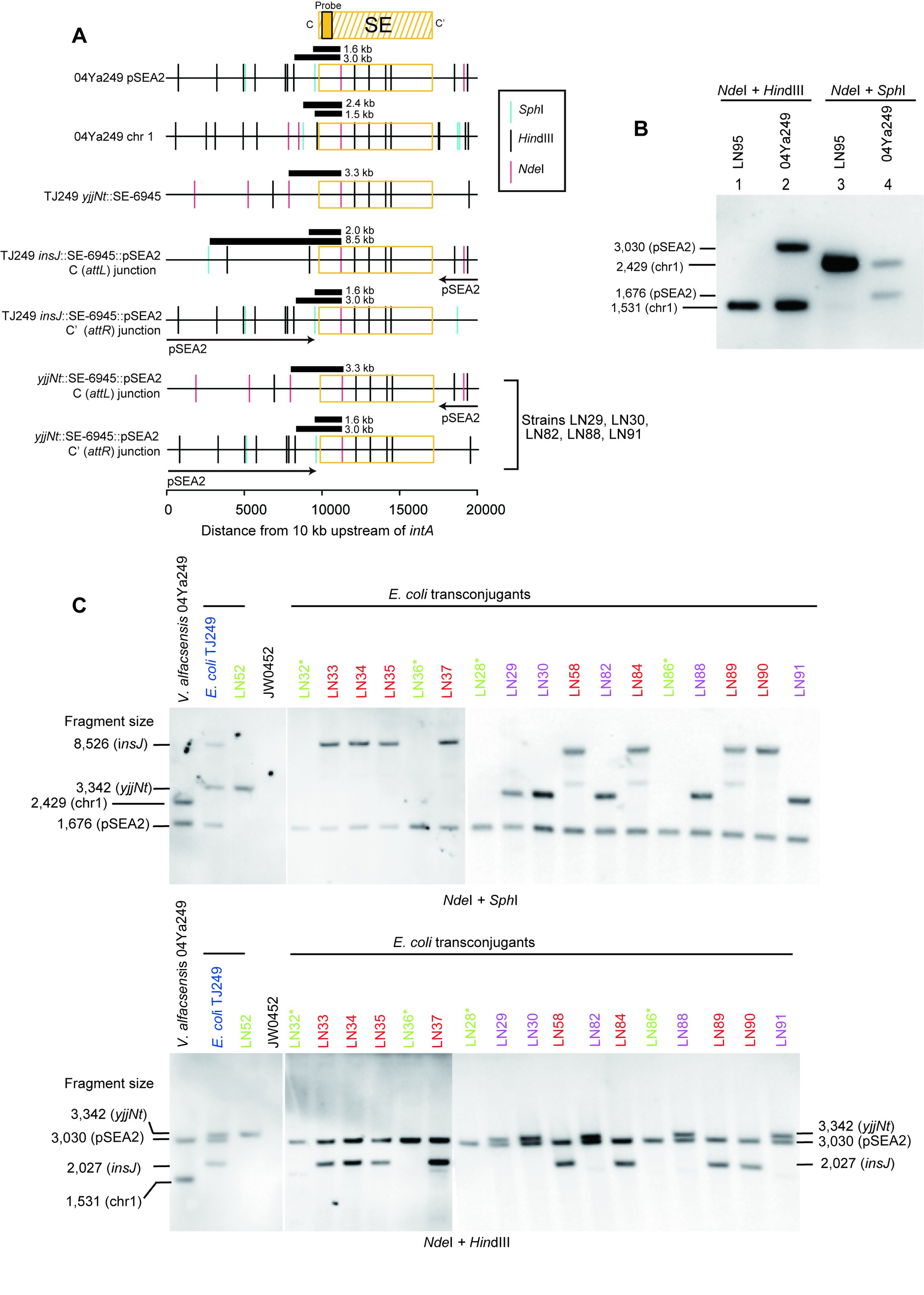

Supplement: S5 Fig — (A) in silico restriction map of DNA segments around SE-6945 integration locations in strain 04Ya249, TJ249, and a hypothetical E. coli strain carrying a pSEA2 insertion into SE-6945 integrated into yjjNt. Locations of SE-6945 and intA probe hybridization region are indicated by the yellow square and black filled square, respectively, above the restriction map. Horizontal black lines indicate the fragments detected by the probe and their sizes. (B) Southern blots of the pSEA2-free Vibrio strain LN95 and the parental strain 04Ya249. Genomic DNA was double digested with NdeI and HindIII (the left two lanes) or NdeI and SphI (the right two lanes). The probe used was 5’ end of intA. Four unique bands originate from fragments shown in the first two rows (04Ya249 pSEA2, 04Ya249 chr1) in panel A. (C) Southern blots of 19 E. coli transconjugants obtained from 19 independent mating assays. Upper panel shows digestion with NdeI and SphI. Lower panel shows digestion with NdeI and HindIII. The color of the strain name indicates the pattern of SE/pSEA2 insertion deduced from restriction maps in panel A: blue, three SE copies (yjjNt::SE-6945, insJ::SE-6945::pSEA2); red, two SE copies (insJ::SE-6945::pSEA2); purple, (yjjNt::SE-6945::pSEA2); lime, one SE copy (yjjNt::SE-6945 or chr::pSEA2); Strains with asterisk (*) are expected to carry pSEA2 in an unknown chromosomal location without generating a SE-6945-chromosome junction. (TIF) [file pone.0271627.s005.tif]

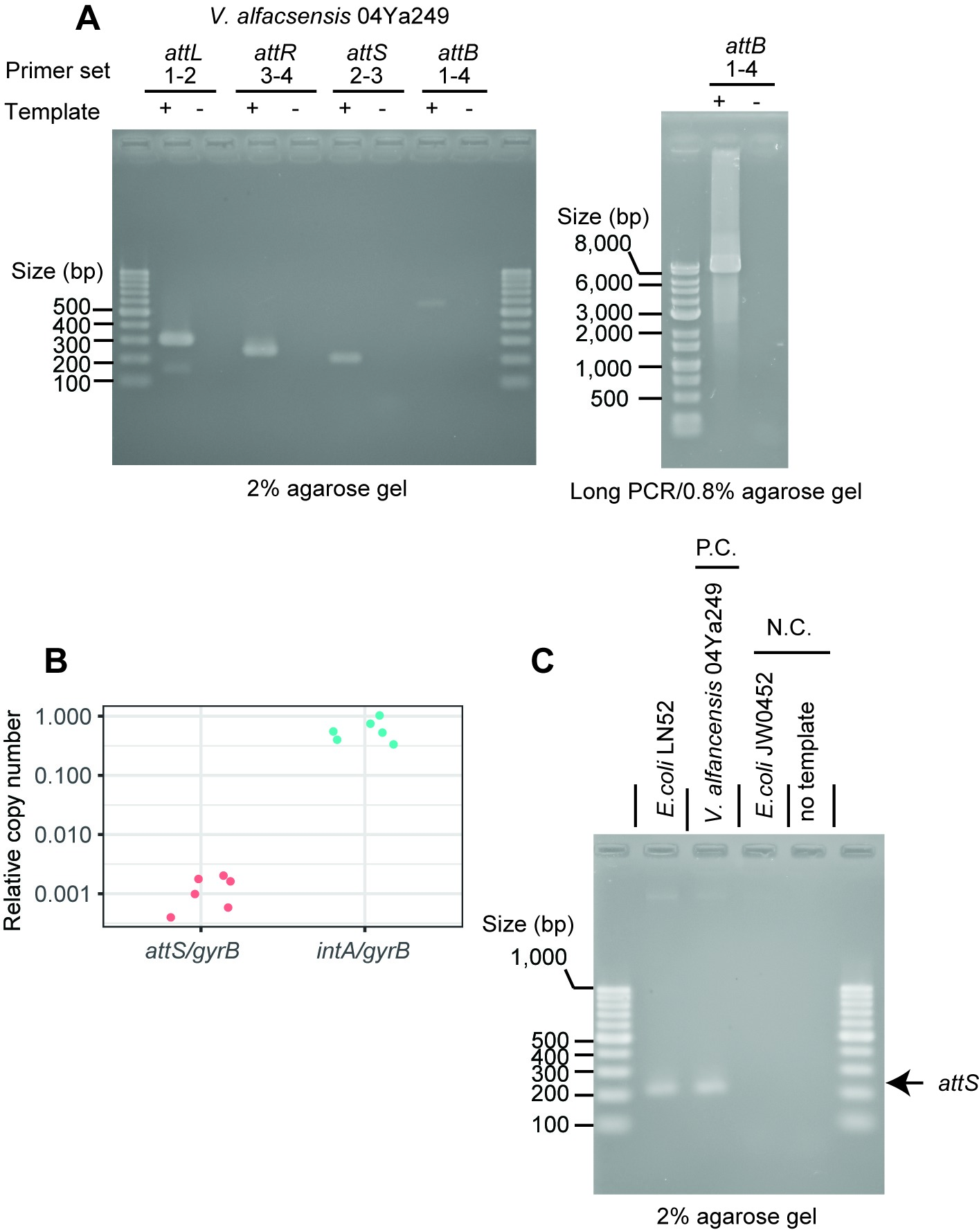

Supplement: S8 Fig — (A) PCR detection of chromosomal attL, attR, attS in 04Ya249. (B) Relative copy number of attS in V. alfacsensis LN95 (no pSEA2). Copy number is represented as relative copy number to gyrB. Primers used are shown in Table 2. (C) PCR detection of attS in pSEA2 free E. coli strain LN52. (TIF) [file pone.0271627.s008.tif]
