# Supplementary material for: Atypical integrative element with strand-biased circularization activity assists interspecies antimicrobial resistance gene transfer from Vibrio alfacsensis
Source: PLoS One. 2022 Aug 2;17(8):e0271627. doi: 10.1371/journal.pone.0271627 (PMC9345347; doi:10.1371/journal.pone.0271627)
Supplement: S3 Fig — Sequences are derived from 04Ya249 genomes (AP019851.1, AP019849.1), TJ239 genome (insJ, yjjNt), and Vibrio harveri strain WXL538 (CP045070.1) carrying a SE-6945-like element carrying a different set of cargo genes. Sequences in red are incorporated into circular copy of SE. 6-bp underlined sequence is incorporated into attS during SE circle formation. 19 bp motif C highlighted in yellow form an imperfect inverted repeat with the motif C′ located the other terminus of SE. Sequence highlighted in cyan is a putative transcription termination motif. Asterisks indicate conserved nucleotides. (DOCX) [file pone.0271627.s003.docx]

Putative *yjjNt* transcriptional terminator

1234567890123456789012345678901234567890123456789012345678901234567890

attL_SE-6945_pSEA2_AP019851.1: TAAAGCCTGATAAAGAAACCCGACAAGTGAACCATCTAATGCAACCCATGGTGCGCCTTTTCATATTGGC 70

attL_SE-6945_Eco(insJ): CTTTCAAGATCCTCAATGCGTCGGTCTTTTGACAGCTCCAATGCTGATGCCGCTTTTTCTGGATCAACTG 70

attL_SE-6945_Eco(yjjNt): TTAATTAAAGGCGTAATTACTTTCTGATAAGGCGAGATTATTAAAGTTGCCATGCAGCGTCCGGGGAAGT 70

attL_SE-6945_Val_AP019849.1: GAAAAAAGAAGATTAAGTTGATGCCAACCCATCGTTGAAAACTATCAACACGATTCAAAAAGCCATGCAG 70

attL_SE-VhaWXL538_CP045070.1: AAGAAGACTAAGTGATGTTCACTGCCAATCATTGTTGAAAGCTATCAACACGATTCAAAAAGCCATGCAG 70

motif C

1234567890123456789012345678901234567890123456789012345678901234567890

attL_SE-6945_pSEA2_AP019851.1: ATATCGCCATGTTTTTCTTACAGCCAACATATCTAAATAGTTGTTTGACATAGATAACAGTATCTGTGTT 140

attL_SE-6945_Eco(insJ): ATATTGCAATGTTTCTTTTACAGCCAACATATCTAAATAGTTGTTTGACATAGATAACAGTATCTGTGTT 140

attL_SE-6945_Eco(yjjNt): GTTGGGCGCTGTTTTTTTTACAGCCAACATATCTAAATAGTTGTTTGACATAGATAACAGTATCTGTGTT 140

attL_SE-6945_Val_AP019849.1: ACAATGCATGGCTTTTTTTACAGCCAACATATCTAAATAGTTGTTTGACATAGATAACAGTATCTGTGTT 140

attL_SE-VhaWXL538_CP045070.1: ACAATGCATGGCTTTTTTTACAGCCAACATATCTAAATAGTTGTTTGACATAGATAATAGTATCTATGTT 140

** * ** * **************************************** ******* ****

Putative start codon for *intA*

12345678901234567890123456789012345678901234567890123456789012345

attL_SE-6945_pSEA2_AP019851.1: TTTATTTGATTTGTTTAGATTGATAGTCTAACTTTAATTTAGATAAGTTAATTGGGTATTGTATG 205

attL_SE-6945_Eco(insJ): TTTATTTGATTTGTTTAGATTGATAGTCTAACTTTAATTTAGATAAGTTAATTGGGTATTGTATG 205

attL_SE-6945_Eco(yjjNt): TTTATTTGATTTGTTTAGATTGATAGTCTAACTTTAATTTAGATAAGTTAATTGGGTATTGTATG 205

attL_SE-6945_Val_AP019849.1: TTTATTTGATTTGTTTAGATTGATAGTCTAACTTTAATTTAGATAAGTTAATTGGGTATTGTATG 205

attL_SE-VhaWXL538_CP045070.1: TTTATTTGATTAAATTAGATTGATAGTCTAACTTTAATTTAGATGAGTGGAATAGGCATTGTATG 205

************ ****************************** *** * * ** ********
