# Supplementary material for: Atypical integrative element with strand-biased circularization activity assists interspecies antimicrobial resistance gene transfer from Vibrio alfacsensis
Source: PLoS One. 2022 Aug 2;17(8):e0271627. doi: 10.1371/journal.pone.0271627 (PMC9345347; doi:10.1371/journal.pone.0271627)
Supplement: S4 Fig — Sequences are derived from 04Ya249 genomes (AP019851.1, AP019849.1), TJ239 genome (insJ, yjjNt), and Vibrio harveri strain WXL538 (CP045070.1). Sequences in red are incorporated into circular copy of SE. The 6 bp underlined sequences originate from the original attB or SE circle, and they are not incorporated into the attS of the SE circle. (DOCX) [file pone.0271627.s004.docx]

motif C’

1234567890123456789012345678901234567890123456789012345678901234567890

attR_SE-6945_pSEA2_AP019851.1: TATTTTGTGTGTAGCCCTTGTGCGTAAAGGGATTCCTAACTTTTTTATCTAACTTTATGTTAAGGGTATT 70

attR_SE-6945_Eco(insJ): TATTTTGTGTGTAGCCCTTGTGCGTAAAGGGATTCCTAACTTTTTTATCTAACTTTATGTTAAGGGTATT 70

attR_SE-6945_Eco(yjjNt): TATTTTGTGTGTAGCCCTTGTGCGTAAAGGGATTCCTAACTTTTTTATCTAACTTTATGTTAAGGGTATT 70

attR_SE-6945_Val_AP019849.1: TATTTTGTGTGTAGCCCTTGTGCGTAAAGGGATTCCTAACTTTTTTATCTAACTTTATGTTAAGGGTATT 70

attR_SE-VhaWXL538_CP045070.1: TATTTTTTGTGTAGCCCTTGTGCGTAAAGGGATTCCTAACTTTTTTATCTAACTTTATGTTAAGGGTATT 70

****** ***************************************************************

1234567890123456789012345678901234567890123456789012345678901234567890

attR_SE-6945_pSEA2_AP019851.1: TTTTTGTTTTCGATGTCACTATTGAGCTTACGAAGAACATCAACATAAGCGGTACCGAGTGAATCGAATG 140

attR_SE-6945_Eco(insJ): TTCTTGGTGCCAATCTTGAGCGCGCGTAAACCAGCTTCTCCGCGCTCTTCATAGACCTTCAGCCACCTGG 140

attR_SE-6945_Eco(yjjNt): TTCTTGTTTCTTAATAATGTGTTGTAAGCCGTAGAAGGCGTGTAGGTCGCACCCTATGCGACCTACACAT 140

attR_SE-6945_Val_AP019849.1: TTCTTGTACTCTAATGAAAAATAGAATTAGGGAAGTTTACAGCCATGCTTATCGTCGTTTCTCCAGCAAA 140

attR_SE-VhaWXL538_CP045070.1: TTTTTGTACTCTAAATAGAAAATAGAATTAGGGAAGTTAAAGTCATGCTCATCGTAGTTTCTCCAGCTAA 140

** *** *

1234567890

attR_SE-6945_pSEA2_AP019851.1: TACACTGGTT 150

attR_SE-6945_Eco(insJ): CTACAGAACC 150

attR_SE-6945_Eco(yjjNt): CAGTCAGGAA 150

attR_SE-6945_Val_AP019849.1: GACACTTGAT 150

attR_SE-VhaWXL538_CP045070.1: AACGCTCGAT 150
