# Supplementary material for: Atypical integrative element with strand-biased circularization activity assists interspecies antimicrobial resistance gene transfer from Vibrio alfacsensis
Source: PLoS One. 2022 Aug 2;17(8):e0271627. doi: 10.1371/journal.pone.0271627 (PMC9345347; doi:10.1371/journal.pone.0271627)
Supplement: S6 Fig — Sequences are derived from strain 04Ya108 (AP024165.1, AP024167.1), E. coli transconjugant TJ108W0 sequenced in our previous study [30], and Vibrio strains carrying SE-6283-like elements listed in Fig 3. Sequences in red are incorporated into circular copy of SE. Incorporation of the 6 bp underlined sequence of TJ108W0 and pSEA1 into attS was demonstrated in the previous study. (DOCX) [file pone.0271627.s006.docx]

motif C

1234567890123456789012345678901234567890123456789012345678901234567890

attL_SE-VroAM7_AP019798.1: CGAAGACCATGCAGCTGCTGACCAGTTCGGCGTGTGGGGCGAGAAAAAGTTTACCCTTAGAAATTTTTTA 70

attL_SE-VscVS-12_CP016307.1: CGAAGATCATGCCGTGGCAGAACAGTTTGGCGTTTGGGGCGAGAAGAAATTTACCCTTAGAAATTTTTCA 70

attL_SE-ValK09K1_CP017919.1: TGAAGACCATGCCGTTGCTGACCAGTTCGGCGTTTGGGGCGAAAAGAAATTTACCCTTGATTATTTTCCA 70

attL_SE-6283_AP024165.1: TGAAGATCATGCTGTTGCAGAACAGTTTGGTGTTTGGGGTGAAAAGAAGTTCACCCTTAGAAATTTTTTA 70

attL_SE-6283_Ecol_TJ108W0: TGAGGACCACCAGGTGTGCGAACAATTCGGCGTCTGGGGTGAAAAGTCCTTCACCCTTAGAAATTTTTTA 70

attL_SE-6283_pSEA1_AP024167.1:TATCTCAAACGATGGCGTCTATGCTATTGATTCTCTTTATGAAGAAGAGGGCACCCTTAGAAATTTTTTA 70

* * * * * ** * ****** ***** *

1234567890123456789012345678901234567890123456789012345678901234567890

attL_SE-VroAM7_AP019798.1: TGACAAAAAACCTTGATGTATTTTTATGACAAACCTATCTTTGATATGTGGGTAACATATCGAGGATTGT 140

attL_SE-VsCVS-12_CP016307.1: TGACAAAAAACCTTGACGTTTTTTTATGACAATTCTATCTTTGATATGTGGGTAGCATATCGAGGATTGT 140

attL_SE-VAlK09K1_CP017919.1: TGACAGCAAACCTTGATTAATTTTTATGACAAAACTACCTTTAATATGTGGGTAACATATTGAGGATTGT 140

attL_SE-6283_AP024165.1: TGACATAAAACCTTGGATTTTTTTTATGACAAAACTATCTTTGATATGTGGCTAACATATCGAGGGTTGT 140

attL_SE-6283_Ecol_TJ108W0: TGACATAAAACCTTGGATTTTTTTTATGACAAAACTATCTTTGATATGTGGCTAACATATCGAGGGTTGT 140

attL_SE-6283_pSEA1_AP024167.1:TGACATAAAACCTTGGATTTTTTTTATGACAAAACTATCTTTGATATGTGGCTAACATATCGAGGGTTGT 140

***** ******** ************ *** **** ******** ** ***** **** ****

Putative start codon for *intA*

123456

attL_SE-VroAM7_AP019798.1: TTTGTG 146

attL_SE-VsCVS-12_CP016307.1: TTTGTG 146

attL_SE-VAlK09K1_CP017919.1: TTTGTG 146

attL_SE-6283_AP024165.1: CTTGTG 146

attL_SE-6283_Ecol_TJ108W0: CTTGTG 146

attL_SE-6283_pSEA1_AP024167.1:CTTGTG 146

*****
