# Supplementary material for: Atypical integrative element with strand-biased circularization activity assists interspecies antimicrobial resistance gene transfer from Vibrio alfacsensis
Source: PLoS One. 2022 Aug 2;17(8):e0271627. doi: 10.1371/journal.pone.0271627 (PMC9345347; doi:10.1371/journal.pone.0271627)
Supplement: S7 Fig — Sequences are derived from genomes listed in Fig 3. Regarding SE-6283, the 6 bp underlined sequences in attR originate from inserting SE circle, and they are not incorporated into attS upon SE circularization. (DOCX) [file pone.0271627.s007.docx]

motif C’

1234567890123456789012345678901234567890123456789012345678901234567890

attR_SE-VroAM7_AP019798.1: AACTCACTGTTAAAGATATAGTTTGTTATAAATCGTCAAAAATTGAGTCTTAAAAAATCCACAAGGGTAT 70

attR_SE-VscVS-12_CP016307.1: AACTCACTGTTAAAGTTAGAGTTTGTTATAAATCGTCAGCAATTGAGTCTAAAAAAATTCCCAAGGGTAG 70

attR_SE-ValK09K1_CP017919.1: AAACCACTATTAAAGAAAGATTTTTCATAAAACTGTCAAAGTTGTCGTCTTAGATTACTTCCAAGGGTGT 70

attR_SE-6283_AP024165.1: AAAATACTGTTAGAGATAGATTTTTCTGCAAATTGTCATAAATTTAGTCTTAAAGAATTTCCAAGGGTAA 70

attR_SE-6283_Ecol_TJ108W0: AAAATACTGTTAGAGATAGATTTTTCTGCAAATTGTCATAAATTTAGTCTTAAAGAATTTCCAAGGGTAA 70

attR_SE-6283_pSEA1_AP024167.1: AAAATACTGTTAGAGATAGATTTTTCTGCAAATTGTCATAAATTTAGTCTTAAAGAATTTCCAAGGGTAG 70

*** *** *** ** * * *** *** **** * **** * * * *******

12345678901234567890123456789012345678901234567890123456789

attR_SE-VroAM7_AP019798.1: TTTGTCATGGGTAAAGTCTACGATGGTCTTCACCGCATTAGCTTCCTAATCAACGAAGA 129

attR_SE-VsCVS-12_CP016307.1: TTCATTATGGGTAAAGTGTATGATGGCTTGCACCGTATTAGCTTCCTAATCAACGAGCA 129

attR_SE-ValK09K1_CP017919.1: AAGTACATGGGCAAAGTATACGACGGTCTTCACCGCATTAGCTTTCTGATCAATGAAGA 129

attR_SE-6283_AP024165.1: GAGGGCATGGGTAAAGTGTACGACGGACTTCACCGCATCAGTTTCCTGATCAACGAGGA 129

attR_SE-6283_ECol_TJ108W0: GAGGGCATGGGCAAAACCTACGATGGCATTCATCGCATCAGCTTCCTGATTGACGCTGA 129

attR_SE-6283_pSEA1_AP024167.1: AAGTTCAAAGGCGTCAAATATGAGTTCGTATACAACGATACTTTCCTCTTTAGCGCTGA 129

** ** ** * * ** ** * * **
