# Supplementary material for: Atypical integrative element with strand-biased circularization activity assists interspecies antimicrobial resistance gene transfer from Vibrio alfacsensis
Source: PLoS One. 2022 Aug 2;17(8):e0271627. doi: 10.1371/journal.pone.0271627 (PMC9345347; doi:10.1371/journal.pone.0271627)
Supplement: S1 Raw images — (PDF) [file pone.0271627.s011.pdf]

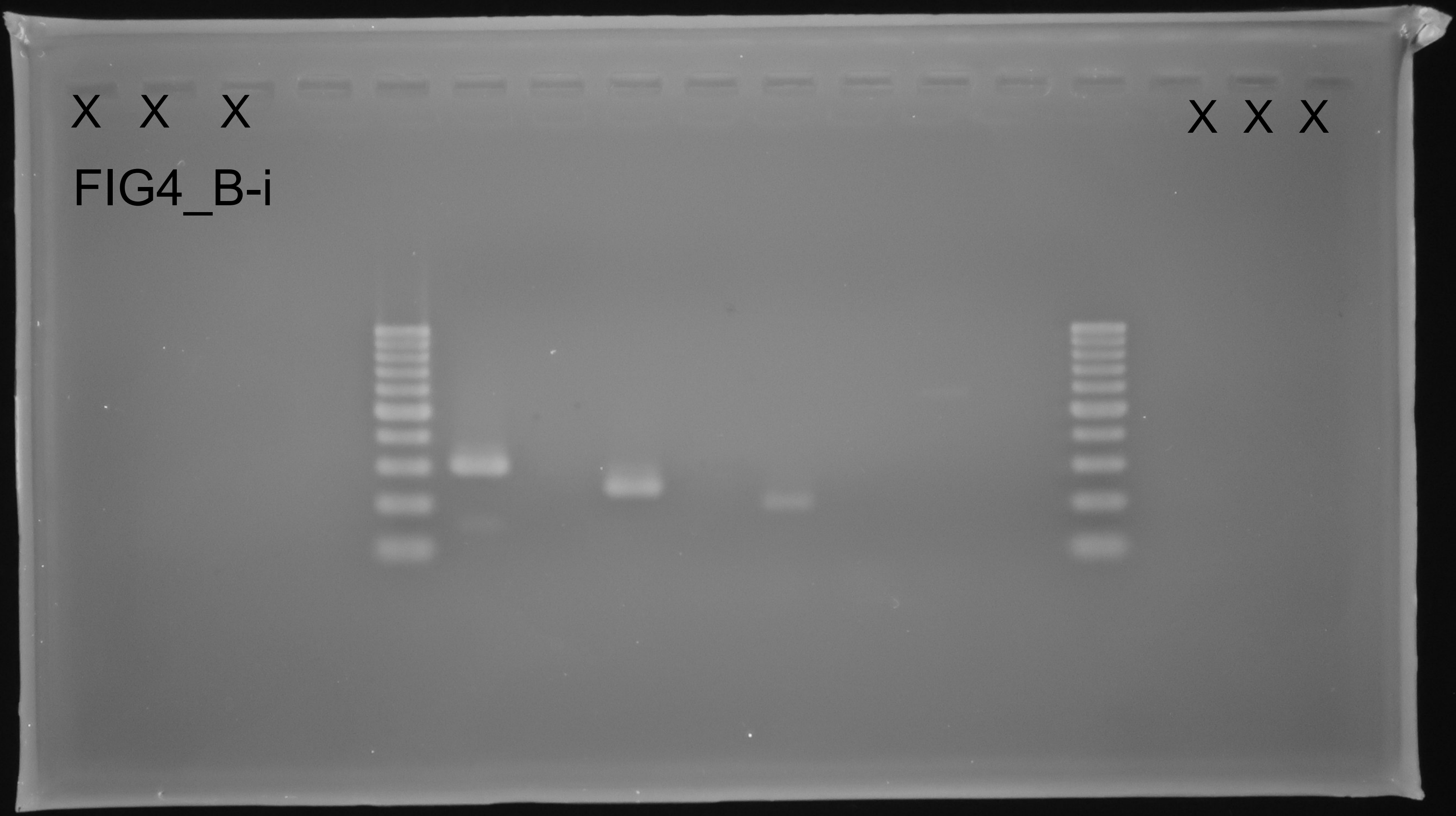
X X X
FIG4_B-i
X X X

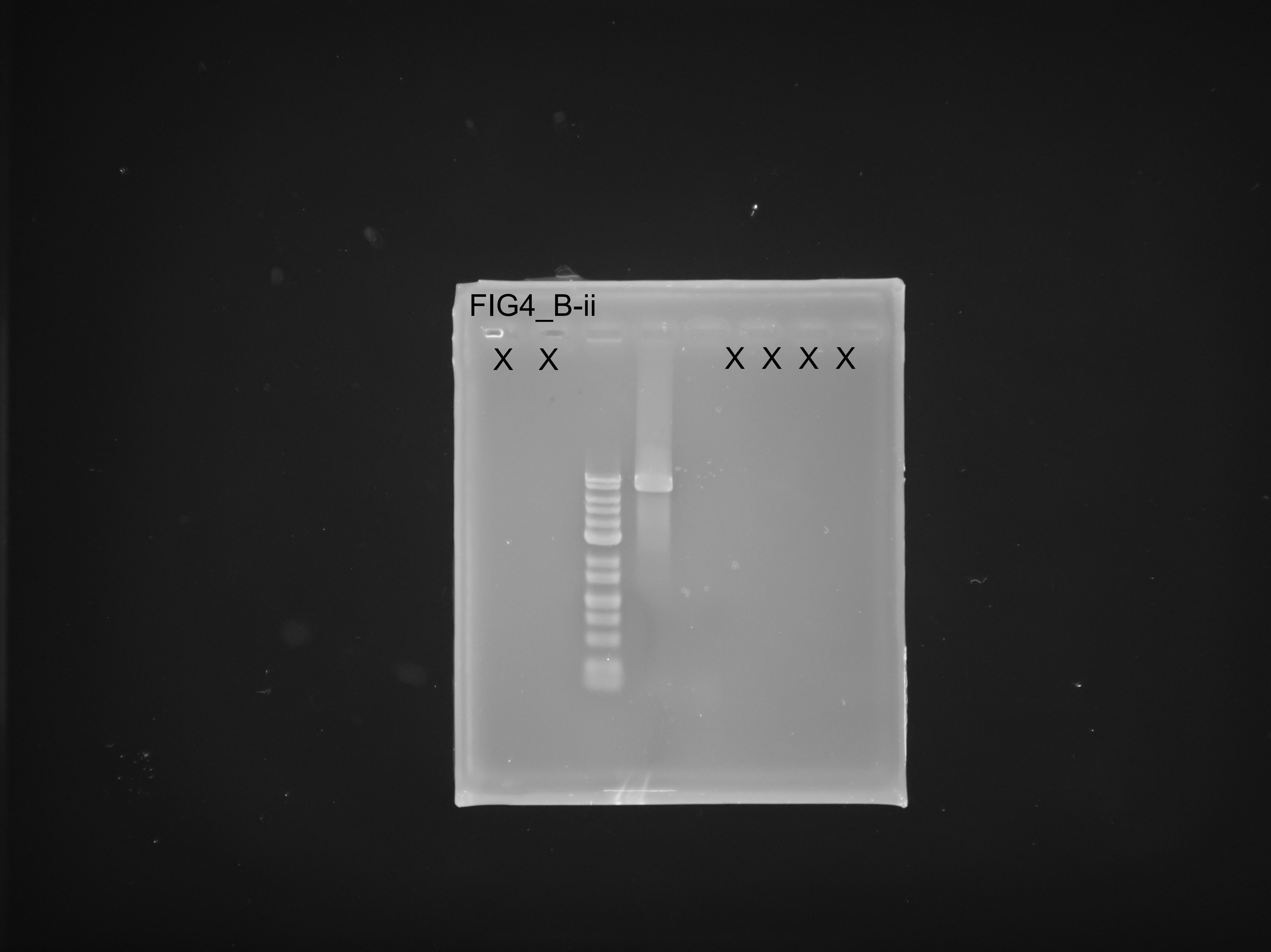
FIG4_B-ii
X X X X X X

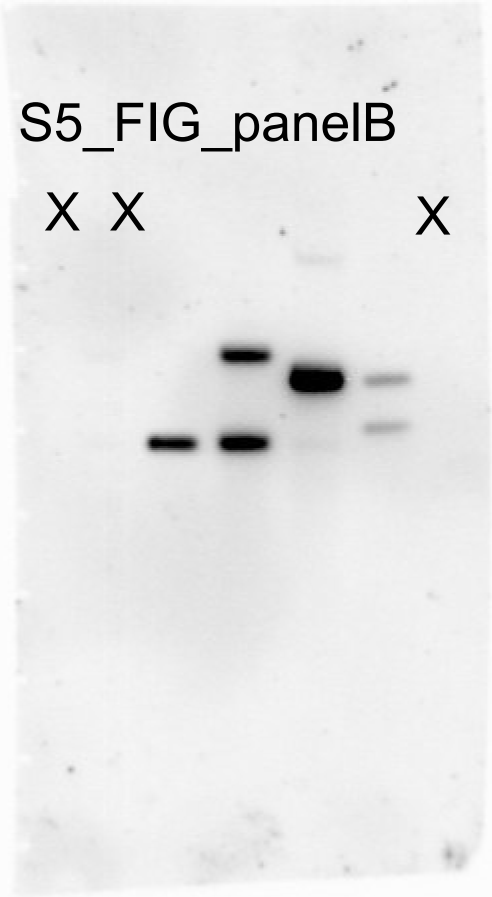
S5_FIG_panelB
X X
X

S5_FIG_panelC_upper-left&lower-left

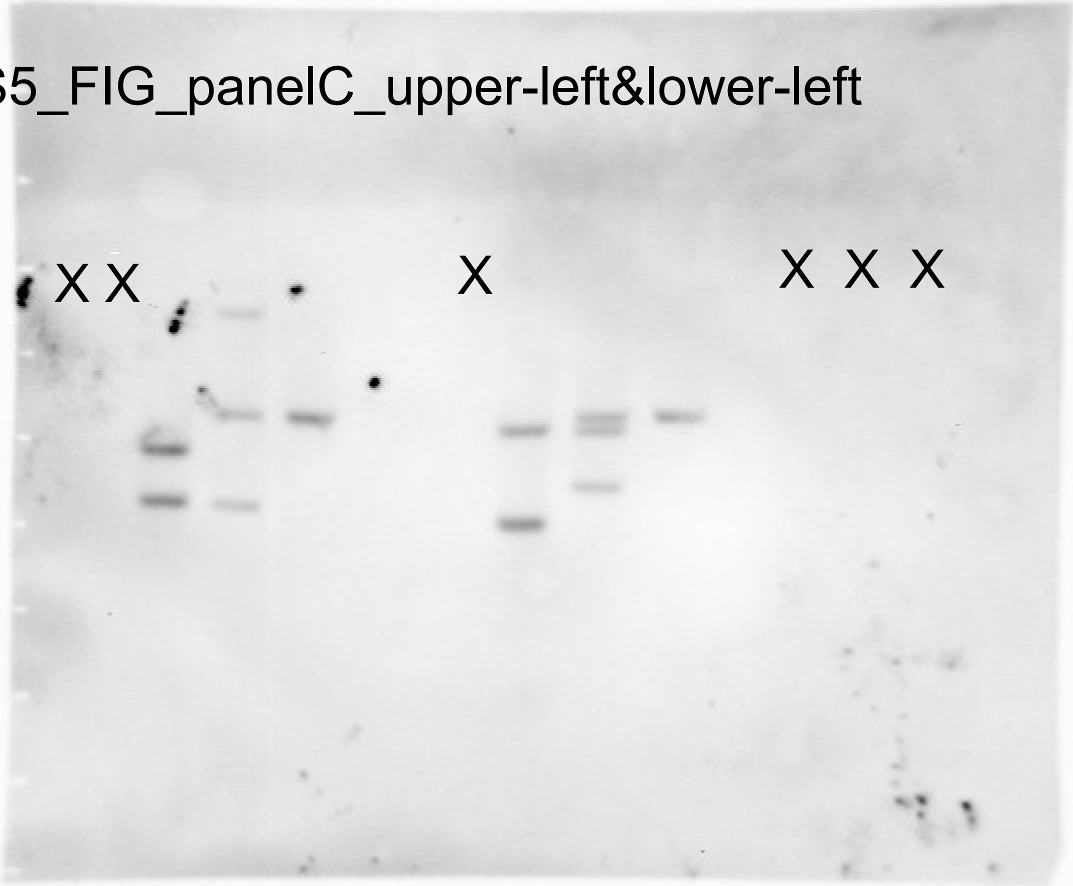

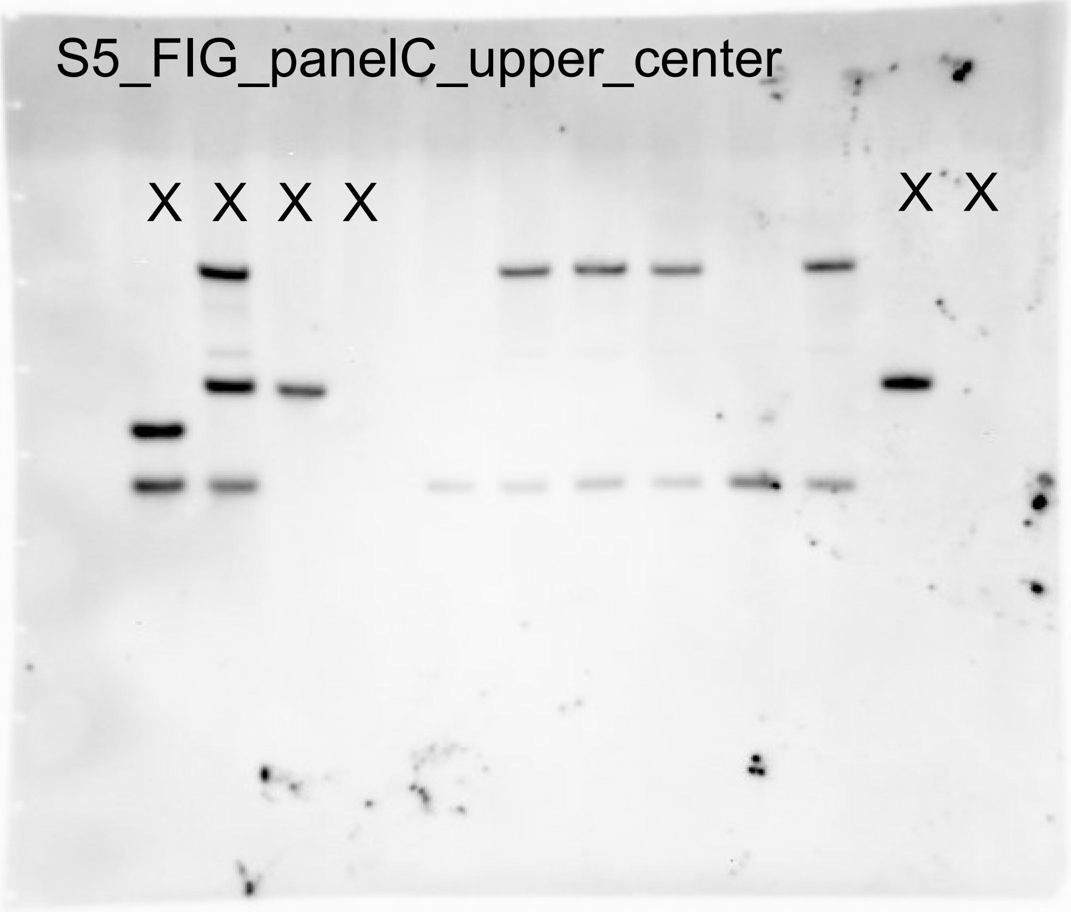
S5_FIG_panelC_upper_center
X X X X
X X

S5_FIG_panelC_upper_right

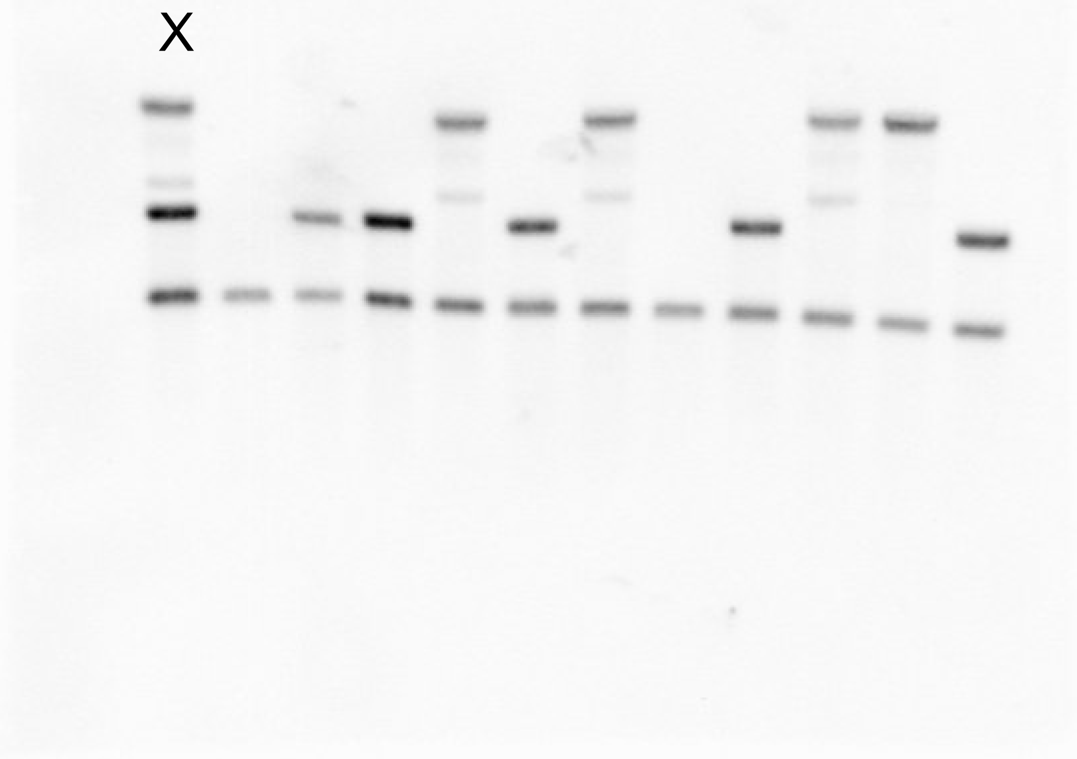

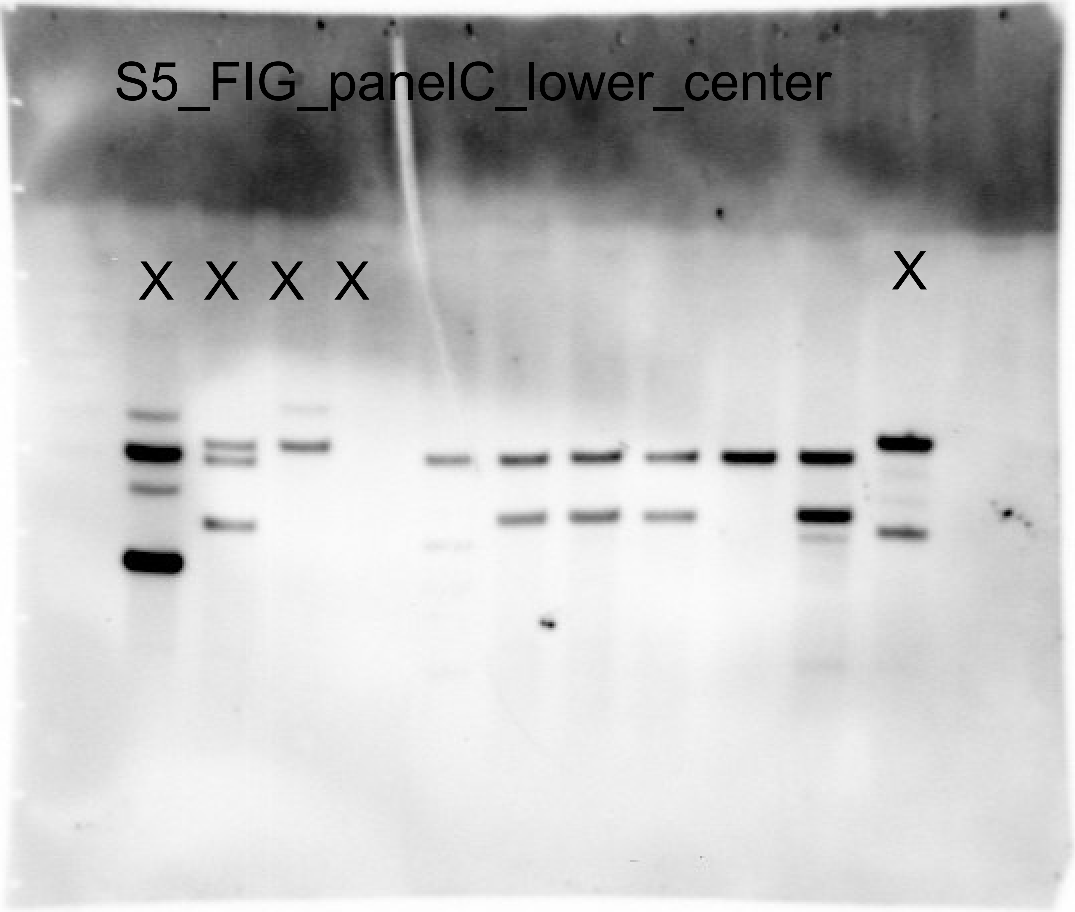
S5_FIG_panelC_lower_center
X X X X
X

S5_FIG_panelC_lower_right

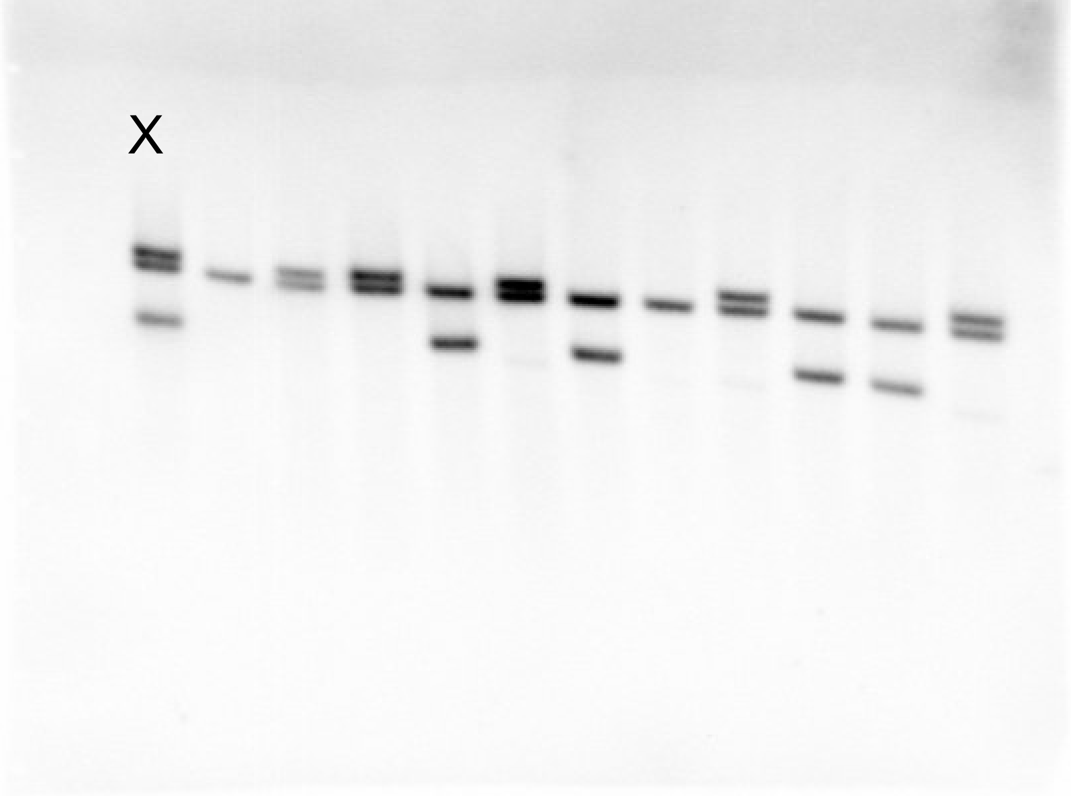

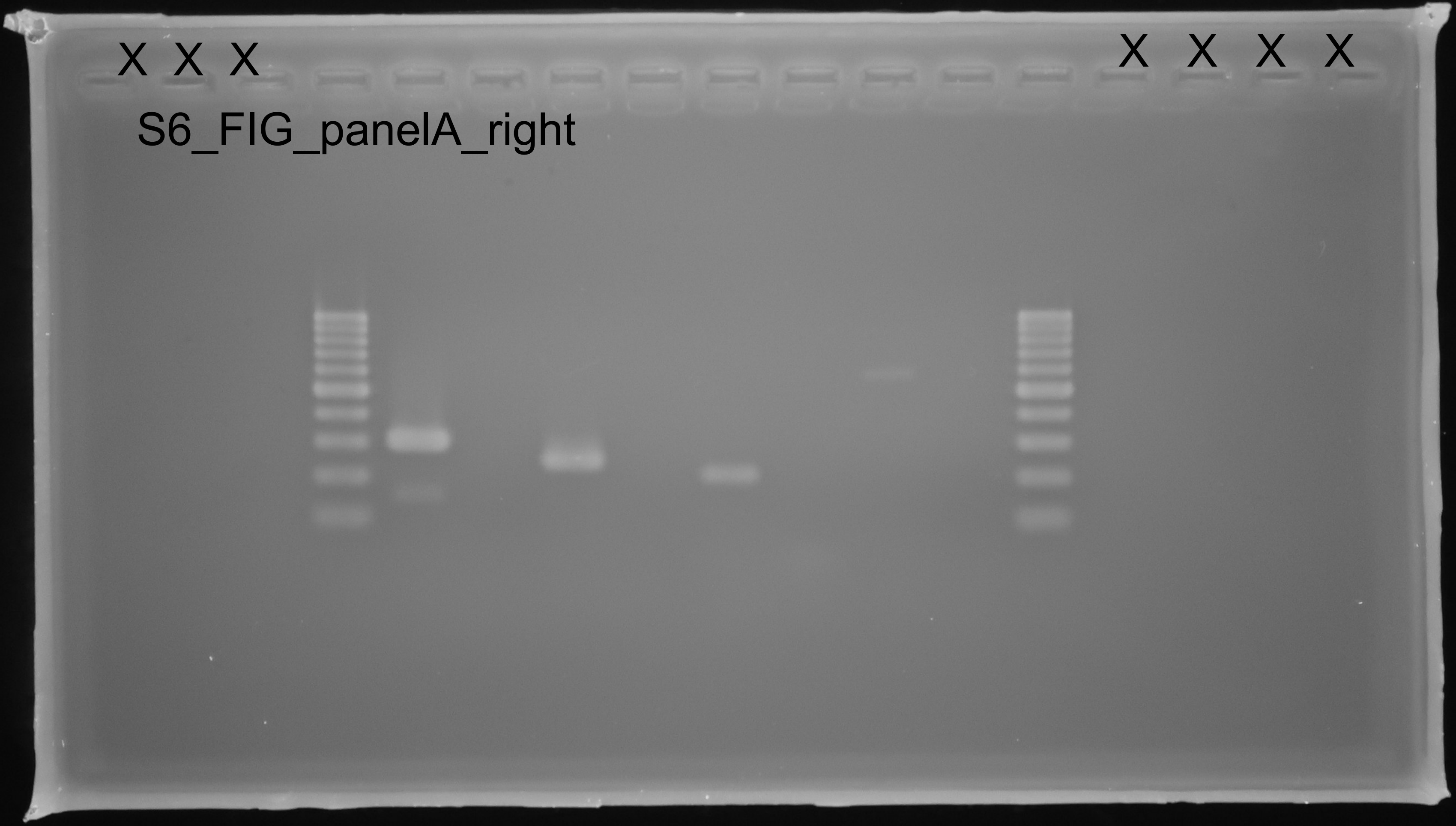
X X X
X X X X
S6_FIG_panelA_right

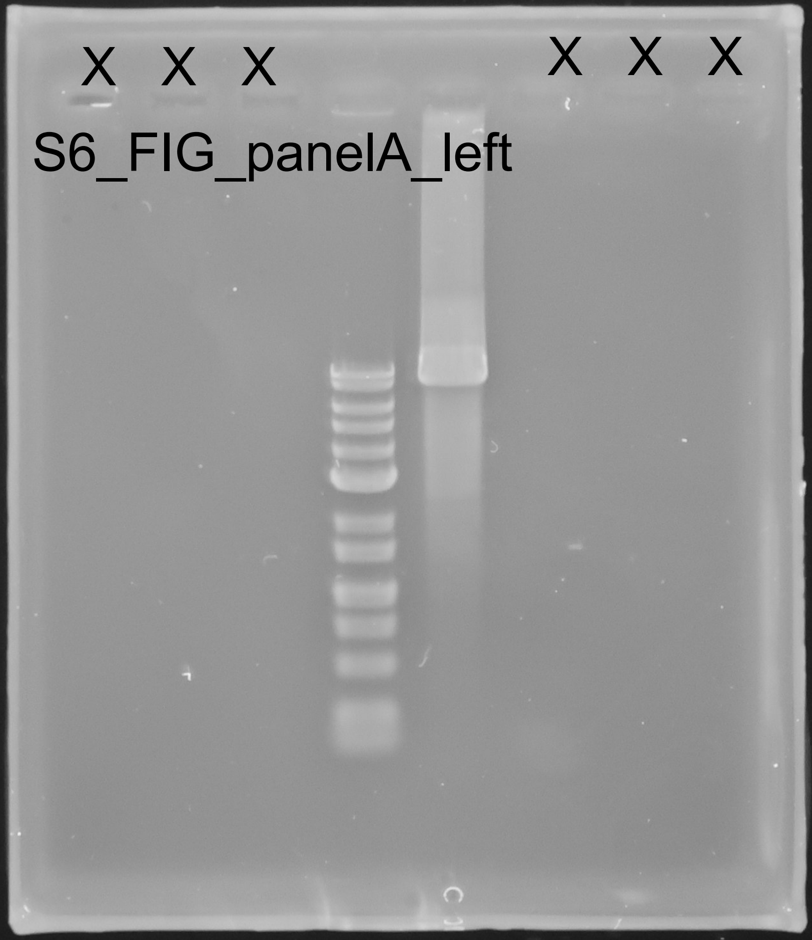
X X X
X X X
S6_FIG_panelA_left

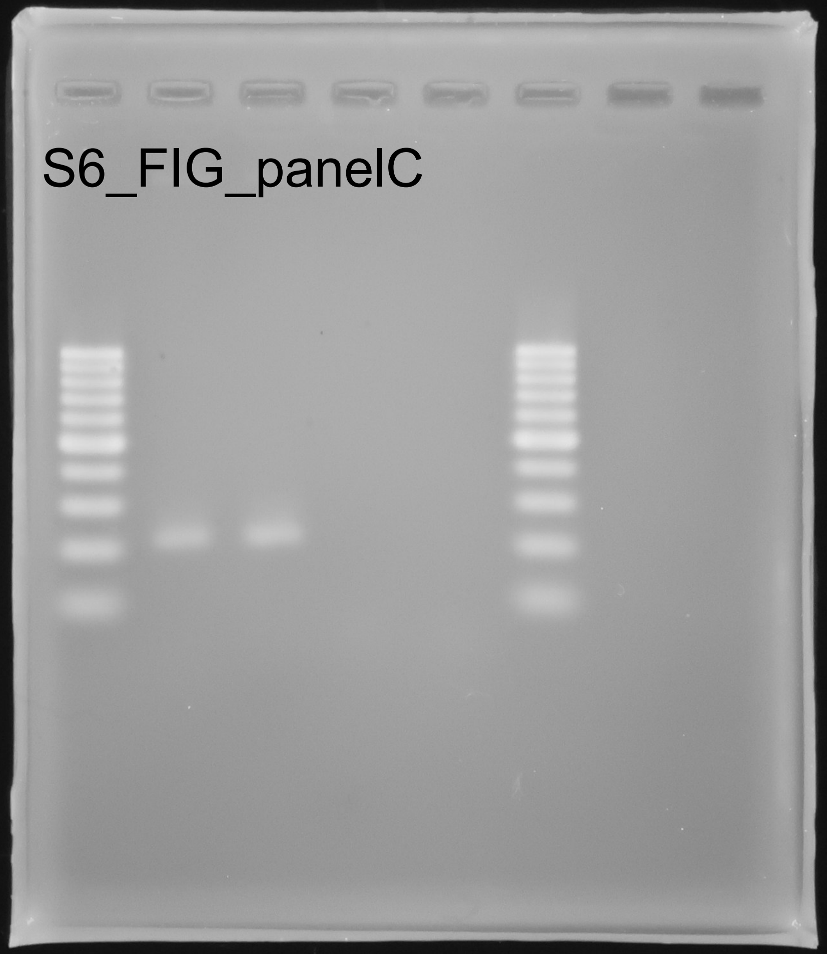
S6_FIG_panelC
