# Supplementary material for: Atypical integrative element with strand-biased circularization activity assists interspecies antimicrobial resistance gene transfer from Vibrio alfacsensis
Source: PLoS One. 2022 Aug 2;17(8):e0271627. doi: 10.1371/journal.pone.0271627 (PMC9345347; doi:10.1371/journal.pone.0271627)
Supplement: Attachment — Submitted filename: Responses_211110LN.docx [file pone.0271627.s012.docx]

Responses to the Editor’s comment

PONE-D-21-18582
Copy-out-paste-in transposition of a Tn6283-like integrative element assists interspecies antimicrobial resistance gene transfer from Vibrio alfacsensis
PLOS ONE

Dear Dr. Nonaka,

Thank you for submitting your manuscript to PLOS ONE. After careful consideration, we feel that it has merit but does not fully meet PLOS ONE’s publication criteria as it currently stands. Therefore, we invite you to submit a revised version of the manuscript that addresses the points raised during the review process.

I have added some comments to help guide in this revision below.

Please submit your revised manuscript by 22-9-2021. If you will need more time than this to complete your revisions, please reply to this message or contact the journal office at [plosone@plos.org](mailto:plosone@plos.org). When you're ready to submit your revision, log on to <https://www.editorialmanager.com/pone/> and select the 'Submissions Needing Revision' folder to locate your manuscript file.

Please include the following items when submitting your revised manuscript:

- A rebuttal letter that responds to each point raised by the academic editor and reviewer(s). You should upload this letter as a separate file labeled 'Response to Reviewers'.
- A marked-up copy of your manuscript that highlights changes made to the original version. You should upload this as a separate file labeled 'Revised Manuscript with Track Changes'.
- An unmarked version of your revised paper without tracked changes. You should upload this as a separate file labeled 'Manuscript'.

If you would like to make changes to your financial disclosure, please include your updated statement in your cover letter. Guidelines for resubmitting your figure files are available below the reviewer comments at the end of this letter.

If applicable, we recommend that you deposit your laboratory protocols in [protocols.io](http://protocols.io) to enhance the reproducibility of your results. [Protocols.io](http://Protocols.io) assigns your protocol its own identifier (DOI) so that it can be cited independently in the future. For instructions see: <http://journals.plos.org/plosone/s/submission-guidelines#loc-laboratory-protocols>. Additionally, PLOS ONE offers an option for publishing peer-reviewed Lab Protocol articles, which describe protocols hosted on [protocols.io](http://protocols.io). Read more information on sharing protocols at <https://plos.org/protocols?utm_medium=editorial-email&utm_source=authorletters&utm_campaign=protocols>.

We look forward to receiving your revised manuscript.

Kind regards,

Ruth Hall, PhD
Academic Editor
PLOS ONE

Additional Editor Comments:

Your manuscript incudes some interesting findings but both reviewers had difficulty following the text and had some concerns about particularly the naming you have used. They have given some quite detailed suggestions all of which look very helpful.

I agree with the reviewers on most points particularly that the text is very hard to follow because the information needed (e.g. sizes of PCR products, predicted sizes of restriction fragments, exactly how PCRs were performed) is not provided.

(Response) Thank you for allowing us to resubmit our manuscript. We sincerely apologize for the inconvenience in interpreting the figures in the previous submission. We found that there were indeed incorrect descriptions about Primers in the Table.

To detect the joint region of the circular form of the integrative element (currently called SE), we showed PCR amplification of 1-kb long *attTn* (currently called *attS*) using an additional set of outward SE primers in the previous submission, as the original outward primers (LN_142_junction2, LN_143_junction2) generate a 206 bp product which was very faint in agarose gel in our laboratory setting. The use of two types of outward primers might have confused the readers.

In this revision, we have improved the electrophoresis condition such that the 206 bp *attS* product is sufficiently clear. Therefore, we removed the information on the 1-kb long *attS* and outward primers used to amplify 1-kb *attS* in the revised manuscript.

Regarding *attB*, we showed only Long PCR results in the previous submission. This may also be the cause for the confusion. In the revised manuscript, we show results of both standard PCR (neither empty *attB* nor occupied *attB* was detected) and Long PCR (only occupied *attB* was detected) in new Fig. 4.

In this revision, we corrected the primer table and updated the figure panel for PCR results.

We added a restriction map panel to the Southern hybridization figure and used more words to explain (i) Southern hybridization results (new S5 Fig) and (ii) *att* sequences (new Fig 4, S3 Fig, S4 Fig): details are provided in the response to the reviewers section below.

We believe that the revised manuscript contains sufficient information to explain the experimental results.

I have a few comments to reinforce or add.
1. Please do not use Tn with a number for an IE. The reviewers have explained why.

(Response) We appreciate your comment and understand the rationale behind your suggestion. However, currently, there are no definite rules proposed for naming newly identified IE, except for established IE groups, such as ICE and IME. In the revised manuscript, we have explicitly stated that the unclassifiable integrative elements, identified in this study, are a new class of mobile elements. Therefore, we have used an “SE-“ prefix (strand-biased circularizing integrative element) to refer to these IEs in the revised manuscript. We believe that using numbers to identify these DNA elements would make it easy to handle (refer to) them in existing and future mobile DNA databases. Therefore, we have used numbers to refer to the SEs in this manuscript.

Regarding the use of the word “transposon/transposition” to refer to the mobile DNA elements, while we are aware that there are scientists who prefer to confine the usage of the term “transposon” to refer to a few classes of mobile elements that use DDE transposase (i.e., IS-composite transposons or unit transposon such as Tn3), the term “transposon” has been explicitly defined as “specific DNA segments that can repeatedly insert into one or more sites in one or more genomes” in Roberts et al. 2008 Plasmid, or “a defined segment of DNA that has the ability to move, or copy itself, into a second location without a requirement for DNA homology” in Curcio and Derbyshire Nat Rev Mol Cell Biol 2003. These definitions share the concept with mobile elements in eukaryotes, and the term transposon is not defined based on the type of DNA strand exchange enzymes used. SEs, handled in this study satisfy these definitions.

ICEs are classified as transposons that move using Y-transposase (tyrosine-recombinase) or S-transposase (serine-recombinase) in a comprehensive transposon review by Curcio and Derbyshire (Nat Rev Mol Cell Biol 2003).

Regarding mobile DNA in prokaryotes, IS91 uses HUH endonuclease, IS607 uses serine recombinase, Casposons uses CAS nuclease for their location changes, but they have all been called transposons.

2. Please do not use “copy-out paste-in”, which is a specific transposition route and not applicable to integration. It’s known that some tyrosine recombinases perform only one strand exchange. Replication is then needed. This route could give the outputs you observe. The first Int system of this type was IntI1 the integron integrase and this was discovered about it in the 1990s. Please consider this.

(Response) We would like to argue with the Editor regrading this point respectfully. “Copy-out paste-in” is the term used in Curcio and Derbyshire (Nat Rev Mol Cell Biol 2003) to classify the type of movements of transposons from a wide range of organisms after the discoveries of ICE, IS91, and IS607, which do not use DDE transposase, though we admit that the word was initially used for the specific IS family. This classification of movements by Curcio and Derbyshire is based on the route/consequences and not based on the proteins used.

While we understand the Editor’s concern, we still think that referring to “copy-out paste-in” is particularly useful to explain the route of transposition of SEs. We illustrated all possible routes of SE circle generation in new Fig 4 A, including “copy-out paste-in,” integron gene cassette excision, and ICE/IME excision. Then we explained how the movement of SEs is different from the known routes. As SEs likely use tyrosine recombinases, they require DNA protein crosslink repair to achieve the IS3-like copy-out (to generate 3′OH). Thus, it is indeed not strictly identical to the movement of the original “copy-out paste-in” transposition of IS3, as pointed out by Reviewer 2.

We also thank the Editor for spending time speculating the movement of SEs. We assume that the Editor talked about the “replicative resolution model” (Escudero et al., Microbiology Spectrum 2015) of gene cassette excision (cut-out of single-strand DNA circle accompanying generation of empty *attC*). The SE circle generation does not accompany the generation of empty *attB*. Therefore, the control of strand exchange is obviously different between Integron and SE. This point is mentioned in the revised manuscript; Line 282-288 in Result and Line 461to 472 in Discussion, and new Fig 4 legend (line 310-333).

We newly cited literature about discoveries of *attI* and 59 bp elements (*attC*) to emphasize the dissimilarity between *attB* of SE and *attI/attC* of Integrons (Stokes and Hall 1989; Stokes et al. 1997), as well as literature relevant to the *attC-attC* recombination (Collis and Hall 1992; Burrus and Waldor 2003; Escudero et al. 2015): line 214, and line 289 in the revised manuscript.

3. Please reduce the use of transposon references and information and replace it with Integrase or IE stories.

(Response) Two transposon papers were removed. However, a few important IS papers are still cited because we think that this research appeals to all audiences studying mobile elements, and we think that dissimilarities (i) between SE and IS, (ii) between SE and gene cassette, and (iii) between SE and ICE/IMEs are all worth stating as emphasized in new Fig 4.

We thank the Editor for reminding us of Integron. We agree that literature relevant to the Integron gene cassette was indeed important but missing. We think that the citation of Integron papers/review (Stokes and Hall 1989; Stakes et al. 1997; Collis and Hall 1992; Escudero et al. 2015), ICEs/IME papers/reviews (Caparon and Scott 1989; Auchtung et al. 2005; Burrus and Waldor 2003; Naito et al. 2016, and others), and CTX phage (Val ME et al. 2005) which are relevant to tyrosine recombinase, and IS (Polard &Chandler 1995; Rousseau et al. 2008) is sufficiently fair. We also emphasized the difference between ICE/IMEs and SEs in line 490-497 in the revised manuscript.

4. Conjugation frequencies must be expressed as per donor. Please fix this. I will accept scatter plots.

(Response) We updated the figure to comply with this (**new Fig 5**).

5. A strain Table is needed.

(Response) We created a strain table (**new Table 1)**. The information on all transconjugants used is included.

6. I was surprised that 2 genes should be called Int as they has such different sizes. So, I checked them using BLAST and found that IntA has the required domain but IntB has no known domains. To see domains, once the search is complete click on the graphic summary and look right at the top. Hence, I insist that this orf be annotated as an orf.

(Response) We appreciate that the Editor showed interest in IntB, and conducted blast searches. As notified by the Editor, at first glance, IntB does not show similarity to known tyrosine recombinase. Homology to Integrase C-terminal domain can be detected by increasing the E-value threshold from 0.01 to 0.05 in Conserved Domain search (which is used by the Editor). The hit subject was cl00213: DNA_BRE_C Superfamily (DNA breaking-rejoining enzymes, C-terminal catalytic domain) in the CD-search. IntB also generated a hit in the Pfam database, and the hit subject was Phage_integrase (PF00589). In this revised manuscript, we made an alignment of IntA, IntB with known tyrosine recombinases, including IntI of R388, using structure-aware prediction program PROMAL3D. We showed that both IntA and IntB possess catalytic (G)-R-H-R-(H)-Y motifs in **new Fig 1B**. The relevant statements are shown in lines 129-137 in the revised manuscript.

6. I also noticed that in the database entry for pSEA2, intA is not annotated as such. Please fix this.

(Response) We have added *intA*, *intB* of (SE-6283, and SE-6945) to the Genbank file of pSEA1 (AP024167), pSEA2 (AP019851), Chromosome 1 of 04Ya249 (AP019850), Chromosome 1 of 04Ya108 (AP024165).

6. IE encoding the same or closely related Int should integrate at the same position. Is your IE in the same gene in Vibrio and E. coli? More on this is needed.

(Response) We appreciate the Editor’s comment. Insertion location of SE-6945 was different between the *E. coli* genome and Vibrio genome (**new Fig 2)**. Interestingly, the insertion location of SE-6283 was the same in the *E. coli* genome and Vibrio genome (**new Fig 6**).

6. The searches I did revealed that there are several IE of this type in Vibrio species. Are they in the same location?

(Response) We conducted additional bioinformatic surveys using SE-6945 and SE-6283 core genes as queries to identify their closely related SEs. We focused on closely related SE because target site preference may be different among distant SE members. We identified new members of SE-6945-related elements and SE-6283-related elements in a different Vibrio species, inserted into the equivalent loci (*bcp* for the SE-6283 related elements; *yaaA* promoter for the SE-6945 related elements) as predicted by the Editor. Due to an increase in the information of the result section, we substantially reorganized figures related to *attB*, *attL*, and *attR*. In the revised manuscript, *attB* of SE-6945 is discussed in Lines 210-216 using **new Fig 2** and **new S3 Fig** and **new S4 Fig,** while *attB* of SE-6283 is discussed in lines 399-415 using **new Fig 6, S7 Fig**, and **S8 Fig**.

We are aware that distantly related SEs are present in the database. We think that the analysis for entire SE members requires a huge amount of bioinformatic analysis, and their target sites are likely different. Thus, we think that those analyses are beyond the scope of this study.

Journal Requirements:

When submitting your revision, we need you to address these additional requirements.

1. Please ensure that your manuscript meets PLOS ONE's style requirements, including those for file naming. The PLOS ONE style templates can be found at

<https://journals.plos.org/plosone/s/file?id=wjVg/PLOSOne_formatting_sample_main_body.pdf> and

<https://journals.plos.org/plosone/s/file?id=ba62/PLOSOne_formatting_sample_title_authors_affiliations.pdf>

2. PLOS ONE now requires that authors provide the original uncropped and unadjusted images underlying all blot or gel results reported in a submission’s figures or Supporting Information files. This policy and the journal’s other requirements for blot/gel reporting and figure preparation are described in detail at <https://journals.plos.org/plosone/s/figures#loc-blot-and-gel-reporting-requirements> and <https://journals.plos.org/plosone/s/figures#loc-preparing-figures-from-image-files>. When you submit your revised manuscript, please ensure that your figures adhere fully to these guidelines and provide the original underlying images for all blot or gel data reported in your submission. See the following link for instructions on providing the original image data: <https://journals.plos.org/plosone/s/figures#loc-original-images-for-blots-and-gels>.

In your cover letter, please note whether your blot/gel image data are in Supporting Information or posted at a public data repository, provide the repository URL if relevant, and provide specific details as to which raw blot/gel images, if any, are not available. Email us at [plosone@plos.org](mailto:plosone@plos.org) if you have any questions.

[Note: HTML markup is below. Please do not edit.]

Reviewers' comments:

Reviewer's Responses to Questions

**Comments to the Author**

1. Is the manuscript technically sound, and do the data support the conclusions?

The manuscript must describe a technically sound piece of scientific research with data that supports the conclusions. Experiments must have been conducted rigorously, with appropriate controls, replication, and sample sizes. The conclusions must be drawn appropriately based on the data presented.

Reviewer #1: Partly

Reviewer #2: Partly

2. Has the statistical analysis been performed appropriately and rigorously?

Reviewer #1: I Don't Know

Reviewer #2: N/A

3. Have the authors made all data underlying the findings in their manuscript fully available?

The [PLOS Data policy](http://www.plosone.org/static/policies.action#sharing) requires authors to make all data underlying the findings described in their manuscript fully available without restriction, with rare exception (please refer to the Data Availability Statement in the manuscript PDF file). The data should be provided as part of the manuscript or its supporting information, or deposited to a public repository. For example, in addition to summary statistics, the data points behind means, medians and variance measures should be available. If there are restrictions on publicly sharing data—e.g. participant privacy or use of data from a third party—those must be specified.

Reviewer #1: Yes

Reviewer #2: Yes

4. Is the manuscript presented in an intelligible fashion and written in standard English?

PLOS ONE does not copyedit accepted manuscripts, so the language in submitted articles must be clear, correct, and unambiguous. Any typographical or grammatical errors should be corrected at revision, so please note any specific errors here.

Reviewer #1: No

Reviewer #2: Yes

5. Review Comments to the Author

Please use the space provided to explain your answers to the questions above. You may also include additional comments for the author, including concerns about dual publication, research ethics, or publication ethics. (Please upload your review as an attachment if it exceeds 20,000 characters)

Reviewer #1: This manuscript describes basic characterisation a non-conjugative integrative element (named “Tn6945”), carrying a beta-lactamase (bla) gene, on the chromosome and a plasmid of a Vibrio ponticus isolate from an aquaculture site in Japan. The element was identified following conjugation with E. coli, when either it or the whole plasmid was transferred to the chromosome, the latter process identified as being dependent on recA. “Tn6945” is related to “Tn6238” previously identified by the same group on a related plasmid in a different Vibrio species. The manuscript seems to imply that “Tn6945” is only the second example of this element to be identified (it would be helpful to make this more explicit if this is the case), in which case describing it would be useful. The approach/results appear technically sound, but it is a bit difficult to tell from the descriptions provided, and the conclusions generally justified, but naming these elements transposons is problematic (see point 1 below). Also, the manuscript requires some reorganization rewriting and condensing, as it not easy to follow and is quite wordy and repetitive in places (see examples below).

(Response) We appreciate the reviewer for showing an interest in this work despite the presence of an incorrect description in the results in the original submission. We understood the reasons why the reviewer was confused. In the revised manuscript, we used more words to explain the Southern hybridization and PCR results and dissimilarity in movements between Tn6945(SE-6945) and Integron gene cassette or ICE/IMEs. Incorrect descriptions about primers and electrophoresis panels have been replaced with corrected versions (details are stated below).

We use the term “*transposon*” based on the definition “specific DNA segments that can repeatedly insert into one or more sites in one or more genomes” (Roberts et al. 2008 Plasmid) while admitting that some scientists prefer to confine its usage to unit transposon (i.e., Tn3, Tn7) or IS-composite transposon encoding DDE transposase. The term “*transposition*” has been defined as “the movement of discrete segments of DNA without a requirement for homology” (Curcio and Debyshire 2003 Nat. Rev. Mol. Cell. Biol), and not based on the type of strand exchange enzyme used. In the introduction section of the revised manuscript, we cited transposon reviews about the definition (Roberts et al. 2008 Plasmid) and transposon classification based on strand exchange enzymes and movement (Curcio and Debyshire 2003 Nat. Rev. Mol. Cell. Biol). In the review, ICE/IME was classified as transposons that use Y- or S-transposase (tyrosine recombinase or serine recombinase). We added these explanations for the terms in lines 53-72 in the revised manuscript.

As Tn6283/Tn6945 are currently unclassifiable into any mobile element classes proposed to date, we coined the term SE (strand-biased circularizing integrative element) to refer to these integrative elements in the revised manuscript. Upon request from the editor, we conducted an additional database survey and found several new members of SEs carrying a different set of cargo genes while sharing only the 4 core genes (**new Fig 2** and **new Fig 6**). Therefore, we think that SEs are worth being proposed as a new mobile element family.

The confusion about the word usage may also stem from the fact that this study needs to handle several distinct intracellular DNA movements using similar or identical words. We, therefore, explicitly stated the rule for the word usage in this manuscript for “transposition,” “integration,” “insertion,” and “translocation*”* in the introduction section of the revised manuscript (lines 96-107). This would help readers regardless of their backgrounds.

1) “Non-conjugative integrative element” vs transposon
Description of “Tn6945”/”Tn6238” elements as a transposons is at best confusing, when they carry tyrosine recombinases rather than true transposases. Inconsistencies/contradictions in the text suggest that the authors are not really sure how to describe them either e.g., “nonconjugative integrative element Tn6283” (line 62), “a new type of transposon” (line 66), “Tn6283-like integrative elements” (line 69), “active integrative element” (lines 115-6) and their is reference to them having both IR (typical if transsponsons) and att sites (typical of integrative elements). While some ICE were historically given Tn numbers it is not helpful to perpetuate such confusion and the nomenclature of these “Tn6945”/”Tn6238” elements needs to be carefully considered and if these are a novel type of element then this needs to be clearer.

(Response) As stated above, our standpoint is that ICE/IMEs should be handled as one class of transposons based on the idea in the review by Curcio and Derbyshire 2003 Nat Rev Mol Cell Biol.

We used the “SE-“ prefix to refer to each SE member in the revised manuscript. We prefer to use 4-digit numbers to keep this compatible with the existing Tn database, and in part considering the ease of handling them in future databases.

We used the terminal *inverted repeat* motifs as a general meaning but not specific TIR of ISs. In the revised manuscript, this phrase was deleted.

2) Lines 111-131 – descriptions of insertions
This section needs to be written more clearly/reorganised/condensed.

Line 111 – this is called “MULTISPECIES: class A beta-lactamase” by NCBI RefSeq. It might be useful to obtain a name for it from NCBI, – “a 7.1 kb repeat region”.

(Response) We requested an update of Genbank annotation files to include gene names suggested by AMRFIndePLuS under “CDS feature”. All AMR-relevant genes in AP019849, AP019850, AP019851, AP024165, AP024166, AP024167 released by now have gene names. “Gene feature” was not amendable by the submitter. It will be automatically updated or added, when necessary, by GenBank.

Line 114 – it might be better to avoid “homology” here? Gene product identity would be higher than coding sequence identity, so this might be written the other way round.

(Response) In the revised manuscript, we showed the Int protein alignment as new **Fig 1B**. Therefore, it is sufficiently clear for the audience that the 4 coding sequences embedded in SE-6283 and SE-6945 are not independently evolved hypothetical proteins but orthologous proteins; thus, using homology (meaning phylogenetic relation) is correct (line 125).

We showed the gene product identify values as follows in the revised manuscript as follows: “gene product identities between SE-6945 and SE-6283 counterparts were very low: 22.5 % for IntA, 20.5 % for CDS2, 23.2 % for IntB, and 26.6 % for CDS4 in blastp” in lines 126-128.

Lines 115-116 – the evidence for this is from the conjugation experiments? This is not clear.

(Response) We used the term “active” based on the evidence for (i) the circular form generation in the absence of pSEA2 in *V. alfacsensis* (**new Fig 4,** previous Fig 3) and plasmid-free *E. coli* (**new S6 Fig**) and (ii) new junction generation (*attL, attR*) in *E. coli* during conjugation experiments. We rephrased this sentence to explicitly state this fact as “This 7.1-kb region was confirmed to be active in generating its circular copy of top strand in *E. coli* and *V. alfacsenis* and inserting it into new target locations in *E. coli* in this study” in lines 129-131.

Lines 120-121 – what is the evidence for targeting, as opposed to random insertion?
(Response). The Southern hybridization experiments (the current **S5 Fig**) were performed to see the target site preference of SE using 19 transconjugants obtained from independently performed mating experiments. However, we forgot to write this motive in the previous submission. In the revised manuscript, we have added a new paragraph regarding the target site preference and have discussed this in lines 181 – 225.

Lines 122-4 – “Tn6945” inserts itself, rather than its IR? It’s not clear whether it is being proposed that “Tn6945” has IR, like a typical e.g., Tn3 family transposon (and why are the repeats called C and C’?) which are internal to such Tn, or it has an att site that recombines with target att site to create hybrid sites, as for integrative elements. att site are mentioned in the Fig. 1 legend but are not discussed until later in the text, which is also a problem.

(Response). We understand the reviewer’s concern. We changed the phrase to “SE-6945 inserts itself ending with 5’-GTA-3 (Fig 1 A) along with an additional 6 bp next to the 5’ end of C at attL on the donor molecule into the target site” (line 177-178).

C and C’ are from core site of site-specific recombination mediated by tyrosine recombinase (i.e. Lambda Int). The problem with this new integrative element is that we cannot deduce how much longer does the cis-element involved in recombination extend from motif C/C’. Therefore, we simply defined the term *attL* and *attR* as “The border regions between SE-6945 and its target locations” and then stated “The border regions between SE-6945 and its target locations were termed *attL* (*intA*-proximal side) and *attR* (*intA*-distal side) (Fig 2 A)” in the revised manuscript (line 169-170). We also elaborated to explain the feature around nicking sites at *attL* and *attR* at lines 170-176 and lines 210-216.

3) Other scientific points
Lines 63, 85, 88, 354, 359 - why was 42 deg C used? This needs to be explained.

(Response) 42 ºC was used to suppress the growth of Vibrio donor strain on the transconjugant selection plate. We have stated this in the Method section in the revised manuscript (lines 567-568).

When we started this research, we did not have rifampicin-resistant mutant of *E. coli*. Therefore, we used 42 ^o^C selection alone to select *E. coli* from the mating mixture. After obtaining TJ249 and its sequencing, we revisited the experiment design, and started using both rifampicin and 42 ºC.

Lines 81 - it might be useful to make it clear when citation are to previous papers from the same group of researchers (i.e., use “We”).

## (Response) In the revised manuscript, we started with “We previously isolated *V. alfacsensis* strain 04Ya249 from sea sediment at an aquaculture site,” as suggested (line 111-112).

## Lines 106, 380-1 – is this version of PacBio alone sufficient to give accurate sequences?

(Response) The oldest reads were obtained in 2016 from PacBioRSII at CLR mode; coverage was > 120x, the chemistry was P6C4 chemistry. Currently, error rate information of that chemistry is not available from Pacbio. However, even under the older setting used in 2013 (<https://www.pacb.com/uncategorized/a-closer-look-at-accuracy-in-pacbio>), the accuracy exceeds QV 60 (99.999%) when the coverage became higher than 25x. Since we obtained 120x coverage, the accuracy of the consensus genome is equivalent to the current HiFi reads or illumina reads assembly. We added the statement “using P6C4 chemistry at Macrogen (Tokyo, Japan) in 2016” in line 623-624 to indicate that these reads were obtained after 2013.

Line 109 – is the accession no. for this given anywhere?

(Response) Accession no. of the genome of 04Ya249 is given in the Accession numbers section. Strain TJ249 is a laboratory strain, and thus we think its genome is not worth registration in Genbank/DDBL/EMBL database. However, its raw reads and assembly are available from figshare (the link is listed in reference in the previous submission) as stated in the method section and new data availability table **new S2 Table**.

Lines 179-80 – att need explaining before this point in the manuscript

(Response) We added explanation for the *att* sites as follows: The border regions between SE-6945 and its target locations were termed *attL* (*intA*-proximal side) and *attR* (*intA*-distal side) (Fig 2 A)” (line 169-170), ”unoccupied target site in the host (*attB*)” (line 173) in the revised manuscript.

Line 223 – what concentration of ampicillin?

## (Response) 100 ug/ml as stated in the Strains, plasmids, and culture media section in Methods: line 553-554.

Line 240 – V. cholerae is not always from sea food?

(Response) *V. cholerae* is indeed not always from sea foods as described in the cited reviews. These descriptions are correct. Thus we did not make changes to the revised manuscript.

Line 250 – the term “super-integron” should not be used. Use e.g., “sedentary chromomsomal integron” instead.

(Response) We changed the super-integron to “sedentary chromosomal integron” in the revised manuscript in line 439.

Line 264-66 – is “Tn6945” the smallest of only two of this type of elements to have been identified to date? Or are there other examples in addition to Tn6283? This needs to be clear, i.e., state in the Introduction that is a new type of element, if this is the case.

(Response) “Tn6945/SE-6945” is the smallest of only two of these types of elements to have been identified to date. In the revised manuscript, we first called Tn6283 as “unclassifiable integrative element Tn*6283”* in line 80*,* then stated that *“*we have termed currently unclassifiable integrative element, SE-6283 and its related elements as a new class of mobile elements: strand-biased circularizing integrative element (SE)” in line 93-95 in the Introduction. In this revision, we show more examples of SEs in new Fig 2 and new Fig 6 based on our new survey.

Lines 266-70 – is it possible to tell anything about how the bla gene might have ended up in this location? Is there really evidence for an “ancient origin”?

(Response) We omitted the relevant sentience in the revised manuscript.

Lines 275-6 – these genes need to be described in Results.

(Response) We appreciate the reviewer’s comment. We explicitly stated that “The biochemical functions of their products were estimated using Pfam and InterProScan in EMBL. The *intA* and *intB* products generated a hit on phage_integrase (CL0382) in Pfam and were further aligned with known tyrosine recombinases using structure-aware alignment program PROMAL3D [32] to confirm the presence of conserved motifs. The *intA* and *intB* products contained the catalytic R-H-R-Y motif (G-R motif in box I and H-R-H-Y motif in box II (Fig 1B)) conserved in tyrosine recombinases [33], though the N-terminal region of IntB is extraordinarily long among tyrosine recombinases. CDS2 and CDS4 products did not generate hits in the Pfam or InterProScan databases.” in lines 133-140.

Lines 278-80 – what is the evidence of this for the “latter” i.e., Tn6283? This is not really clear from the Results and both references are about IS.

(Response) We used more words and figure panels to explain the results in the revised manuscript (lines 283-296, lines 304-308, lines 342-359; new **Fig 4 panels A, D, E,** new Fig 4 legend). We believe that **new Fig 4** would help readers understand our interpretation: the SE’s movement is more like IS than Integron gene cassette or ICEs.

The previous reference for the IS911 review indeed stated that in its Fig 5B, “*cleavage of the left or right inverted repeat (IR) and attack of the other end”,* and it generates a single-strand DNA bridge: this means that both strands can be nicked, but they do not happen simultaneously, and a single-strand bridge is generated using the top or bottom strand. In the previous reference for the IS (Kosek et al 2016, Figure 2 C) indicates the presence of top strand exchange and bottom strand exchange products, and there is a statement “*This suggests that TnpA can use either TIR to strand transfer into the other*”.

For clarity, we changed the cited articles to Polard 1995, which focused on the reaction of single stand bridging (figure-eight formation) (Polard 1995) and the bias of two IRs of IS911 (Rousseau 2008). We think that current references are more appropriate to argue about the presence of strand bias of single-strand DNA bridging.

Lines 292-307 – a table explaining the different strains, with some additional details in the text in the case of more complex constructions, might be clearer. The first sentence should be deleted and the citations included in other places.

(Response) We made a new Table of strains and plasmid list as **new Table 1**. SE-insertion location in each strain is shown in the new **Table 1**. The first sentence was replaced with “Strains and plasmids used in the study are listed in Table 1” (line 508).

Line 347-59 – as some strains used are transconjugants, it might be more logical to describe conjugation experiments earlier in Methods? Line 355 is ambiguous and needs to be written more clearly.

(Response) The section on the conjugation method was moved to an earlier position in the revised manuscript. Then, we have described the details of the selection conditions and motives of the experiments in lines 567-579.

Lines 364-8 – digestion should be described before hybridisation and something like “using the 5'end of intIA, amplified using primers LN112 and LN113 (Table 1) and the PCR DIG… as probe” would be simpler.

(Response) We thank the reviewer for this suggestion. We changed the order of phrases and then added more explanation for the method in the revised manuscript (line 604-605)

4) Minor/formatting/wording/English etc
Some streamlining/reducing repetition in different sections etc. is needed, as well as some reorganization. Some examples are given below.
Line 61 – “The” is not needed here.

(Response) “The” was deleted in the revised manuscript. Line 79.

Lines 63-4 – suggest “…, following conjugation it could integrate into the chromosome by…”

(Response) We rephrased that sentence as suggested: “Although pSEA1 could not replicate in *E. coli* at 42 °C, following conjugation, it could integrate into the *E. coli* chromosome by homologous recombination between two Tn*6283* copies: one on pSEA1 and another that moved from pSEA1 into the chromosome”.

Line 71 – “in this study we mated”

(Response) We removed ‘,’ as suggested. Line 89.

Lines 84-91 are unclear and need rewording.

(response) In the revised manuscript, we split descriptions of the conjugation experiments for three different purposes into three distinct sections (line 111, a section starting at line 182, a section starting at line 228). Now, this paragraph only describes the experiment used to isolate TJ249. We hope that this has made it more clear.

Lines 110 etc – change “beta” to beta symbol

(response) the beta of beta-lactamase was changed to beta (β) symbol in the revised manuscript (line 28; line 110; line 122; line 124; line 889)

Lines 118, 120 etc – suggest present tense for facts that are still true i.e., “contains”. “indicates” etc.

(response) We appreciate the reviewer’s suggestion. We modified the text as suggested. Lines 161 and line 167 in the revised manuscript.

Line 125 – “the previously reported plasmid” can be deleted.

(response) We deleted “the previously reported plasmid” in the revised manuscript (line 155).

Line 127 – can delete “that of”.

(response) We deleted “that of” in the revised manuscript (line 157 in the revised manuscript).

Line 128 – could be condensed to “…from Southern hybridization (19)”

(response) We deleted “in our previous study” as suggested (line 158).

Lines 129-30 – suggest “carries Tn6945 in the same place as in…”

(response) We used “carries SE-6945 in the same two locations as” in the revised manuscript (line 159-160).

Line 134 – “pSEA2” would be clearer than “the plasmid” here. Also line 143 – “integration of pSEA2”

(Response) We used “pSEA2” as suggested in line 229 in the revised manuscript. A phrase corresponding to the previous line 143 no longer exist in the revised manuscript.

Line 136 – “homology” is not really the best term here, if they are identical?

(Response) We use a more direct expression in the revised manuscript as follows: “by homologous recombination between SE-6945 copies: one on the chromosome and the other on pSEA2” in the revised manuscript”. Line 230-231.

Line 142 – suggest “movement of Tn6945 to new locations”

(Response) Homology independent location change of DNA segment is actually identical to the term “transposition” as stated in the Curcio and Derbyshire review (2003), regardless of the strand exchange enzymes used. We wish to follow it (for example, line 226; a phrase corresponding to the previous line 142-143 no longer exist in the revised manuscript). For clarity, the rules of word usage in this manuscript are described in the Introduction of the revised manuscript (line 96-line107).

Line 184 – meaning actually “chromosomal copy of Tn6945 in V. alfacsensis is functional”?

(Response) Because only one SE-6945 copy is present in LN95, detection of circular SE-6945 in LN95 indicates that the *cis*-elements (*attL/attR*) and strand exchange enzymes encoded in the chromosomal SE-6945 is indeed functional, while those of SE-6945 on pSEA2 is obviously functional as its transposition was observed in E. coli.

We explicitly stated that “This suggests that the *cis*-elements (*attL*/*attR*) and strand exchange enzymes encoded by the chromosomal copy of SE-6945 are also functional in *V. alfacsensis*” in lines 304-306 in the revised manuscript.

Line 198 – suggest “two sequences” rather than “sequence types”

(Response) We used “two sequences” as suggested in the revised manuscript (line 349).

Line 199 – “absence of pSEA-2 from”

(Response) We are afraid that we could not find the relevant points in the manuscript. We used more words to explain the strand bias in *attL x attR* recombination: “These two types of spacer sequences can arise from both pSEA2 and chromosome 1 (Fig 4D). However, only one sequence (*attS* type 1) was detected in the pSEA2-free strain LN95 (Fig 4E), indicating that strand exchange occurs only on the top strand in *attL* × *attR* recombination on chromosome 1 (Fig 4C). The occurrence of *attS* type 1 and *attS* type 2 in 04Ya249 (Fig 4E) agrees with the top strand exchange in *attL* × *attR* recombinations on chromosome 1 and pSEA2, respectively (Fig 4D).” We believe that these words would suffice: lines 351-357.

Line 206 – “a” of attB needs to be italicised

(Response) We corrected *attB* in line 336.

Lines214-5 – ambiguous and needs rewording, “respective” is probably not needed (nor line 219)

(Response) We deleted “respective”. We also clearly stated the observed pSEA2 integration pattern in all transconjugants in a new paragraph in line 181-199 in the revised manuscript. The sentences are now as follows: “Southern hybridization analysis of other JW0452 transconjugants revealed one to two copies of SE-6945 in their genomes (S5 Fig). Location of SE-6945 insertion became evident based on Southern hybridization for 15 among 19 transconjugants” (line 376-377).

Line 225 – “was highest”

(Response) We deleted “the” in the revised manuscript (line 383).

Line 229 – better to say something like increased copy number resulted in increased resistance?

(Response) We rephrased the sentence as suggested (line 386-387). In this revision, we elaborated to explain the Southern hybridization results. We coincidently found that insertion location also affects MIC (**new S1 Table**). A new statement was added at the bottom of this section as follows: “MIC of individual strain is shown in Table S1. When focused on a class of MIC 500, there was a trend that strains with SE-6945 insertion into insJ show a higher MIC than strains with insertion into yjjNt. Thus, the location of SEs can also affect phenotype variation, possibly due to the differences in the expression levels of their cargo genes.” (lines 388-391)”.

Line 247 – suggest “other conjugative”, might be more logical to mention islands after all of the plasmids?

(response) We have placed ICE and IME side by side in the manuscript (line 438).

Lines 347-8 – giving the strain names here is not really necessary.

(Response) Strain names were deleted (line 559).

Lines 393 – this information might be more easily accessible if presented in a supplementary table, with the most relevant accessions quoted in the text.

(Response) We changed the paragraph title from accession numbers to Data availability (line 642). The accession numbers are summarized in the **new S2 Table** in the revised manuscript.

References – several references are missing page nos. (e.g., Refs 5, 8, 15, but all need checking) and Title Case (i.e., First Letter of Each Word In Upper Case Like This) should be removed ((e.g., Refs 6, 8).

(Response) We apologize for the inconvenience. We corrected the page numbers and modified the title case to be consistent.

5) Figs.
These could be improved/simplified/split into smaller figures, considering how they would look on a journal page and if they relate to different parts of text.
Fig. 1 - I don’t think that part A is necessary – the information in it is clear from the text. If retained then the brackets need to be explained. Part B(i) is not clear (e.g., the legend does not explain what “CAIM” is and inverting the orientation of pSEA2 would make the comparison clearer) and is probably not needed. I would suggest putting Part C first, then B(ii), with a line underneath showing pSEA2 and then part D.

(Response) We have deleted panel A. Genome comparisons figure (panel B) was incorporated to **new Fig.2,** while attL attR figure panel (previous panel C/D) was moved to **S3 Fig, S4 Fig, Fig 4,** due to increase in related data. We use **new** **Fig 1** just for explaining the genetic features of SEs.

Fig. 2 could be supplementary. It is not clear what “divided by the donor CFU and recipient CFU” in the legend and “Log(transconjugant/(donor/*recipient)” on the Y axis mean?

(Response) Showing transfer frequency by log (T/(RxD)) has been becoming more popular (<https://www.ncbi.nlm.nih.gov/pmc/articles/PMC3687034>; <https://www.frontiersin.org/articles/10.3389/fmicb.2020.02070/full>)

as its data distribution becomes more like a normal distribution, and thus parametric tests (such as ANOVA) can be applied when comparing data. As our research does not really require a parametric test, we used a traditional “T/D” presentation per request from the Editor in the revised manuscript.

Fig. 3B and C could be supplementary. In Part A the Chr2, pSEA and pYa249 diagrams are not really needed and maybe the remainder could be combined with Fig. 1C, B(ii) and D?

(Response) We moved the gel image for strain 04Ya249 and qPCR result to supplementary material (new **S6 Fig**). We updated the figure panel of PCR design to explain the copy-out model in more detail (new Fig 4). As we found the attL, attR sequence useful when explaining the copy-out model, we combined previous Fig. 1 D with the PCR experiment result and design, then generated new **Fig 4 panel C and D** in this revision.

Supplementary Information
The order of Figs S1 ad S3 seems to have been switched vs. the legends.

(response) We apologize for the incorrect descriptions. We confirmed that Figure legends and supplementary figures match in the revised manuscript.

It would be helpful for review if the legends were on the same page as each figure.

(Response) In the PLOS authors guide, it is stated that “List supporting information captions at the end of the manuscript file. Do not submit captions in a separate file”. Therefore, we did not add legends in Supplementary figures.

Reviewer #2: The manuscript describes the identification of an interesting integrative element containing a bla gene from marine Vibrio. It is detected in chromosomal and plasmid sites and it's transfer and integration into E. coli is demonstrated. These are notable findings. Unfortunately, as noted below, I feel there are a significant number of issues that need attention.

Lines 1 (manuscript title), 133, 135, 164 ,166 and 203 (and possibly elsewhere): To avoid confusion, it would be preferable for movement (translocation) of Tn6945 NOT be described as "transposition"; it is site-specific integration, and it doesn't involve a transposase.

(Response) As stated above, we wish to use the terms of *transposon*/*transposition* defined by mobility of DNA segments and not by the type of DNA strand exchange enzymes, following the published reviews (Robers et al Plasmid 2008, Curcio and Derbyshire Nat Review Mol Cell Biol 2003), while we understand that some scientists prefer to confine their usage to a few families of transposable elements in prokaryotes.

Furthermore, particularly in this work, the use of the term “integration” to refer to “location change” is incorrect in that *integration* is just the last step of the location change, and it would rather confuse the readers.

The term *translocation* has been used to refer to the rarely occurring location change of chromosomal segments in organisms carrying multiple chromosomes, and thus is not suited to refer to the repeatedly occurring movements of mobile DNA mediated by a sequence-specific strand-exchange enzyme.

Therefore, in the revised manuscript, we first mentioned the word definition and transposon classification by citing reviews (Robers et al., 2008 Plasmid.; Curcio and Derbyshire 2003 Nat Rev Mol Cell Biol): ICEs are classified as transposons that use Y- or S-transposase in Curcio and Derbyshire 2003), then we mentioned rules of term usage in this manuscript at the end of introduction section (line 94-105).

We believe that these changes would help all readers who have different images for the word “transposon,” including readers studying genetics in eukaryotes. The current tile no longer contains “transposition” or “movement”.

Line 75: "...embedded on the plasmid..." should be "...embedded In the ELEMENT...".

(Response) One AMR gene (*bla*) is embedded in SE, but other AMR genes were embedded outside the SE. Those AMR genes outside the SE-6945 are also integrated into *E. coli* (which is out of the host range of plasmid) using homology of SE-6945. In the revised manuscript, the relevant phrase was deleted. But we still think that our previous description was correct.

Line 87: Selection said to include tetracycline and ampicillin, yet next sentence states transfer frequency for tetracycline only. How can this be?

(Response) In the revised manuscript, we stated “Tc Ap resistance” instead of “tetracycline resistance” in line 243.

Line 95, Fig 1Bi legend: More description is required - why is CAIM 1831 segment shown? Doesn't actually show "insertion sites", rather comparative locations of Tn6945. This figure doesn't seem to provide any useful information beyond what should be in the text (see below) and hence should be removed.

(Response) We understand the reviewer’s concern. In the revised manuscript, we deleted CAIM 1831 genome from the insertion location comparison figure panel (new **Fig 2 A**), and showed only its unoccupied *attB* in new **Fig 2B**.

Line 109: S1 Fig is cited but the legend for S1 is incorrect. The provided S3 legend corresponds to the figure I think. That legend needs to provide the definition for Crick/Watson strands used here since various exist. Is there a meaningful reason why Chr1 of 04Ya108 is shown inverted with respect to Chr 1 of the other strains? If not, it should be reverse complemented for consistency.

(Response) We corrected the legends accordingly. We added the following modification in the legend in the revised figure legend: “Purple dots indicate a match on the Watson strand (5’ to 3’ on the top strand in Genbank file), and light blue indicates a match on the Crick strand (5’ to 3’ on the bottom strand)”: line 873-874 in the revised manuscript.

We arranged Genbank annotation files such that the dnaA position comes first and coded on the “Watson Strand”. There is a small inversion around the dnaA gene in 04Ya108; therefore, a large portion of chromosome 1 was inverted relative to the dnaA region and this may be the reason why the reviewer felt that our presentation is “not consistent”.

We did not make changes on the fasta files used in data analysis is to prioritize the reproducibility of results. But we added arrows to indicate the position of SE-6945 such that the reader can easily follow the insertion locations in the inverted chromosome (**new S1 Fig**).

Line 112: Rewrite sentence to include that the segment is absent from the corresponding region of CAIM 1831.

(Response) We deleted CAIM 1831 from Fig 1 (relevant to previous line 112) due to major changes in the genome comparison section (**new Fig 2** and lines 119-122). We simply used sequence of CAIM 1831 to show unoccupied target sites (*attB*) in new Fig 2B.

Line 114: Indicative values should be provided for "very low gene product identity".

(Response) We added the identity values in the revised manuscript as stated above.

Line 115-117: It is not clear to me what data specifically has "confirmed" that the repeated region is an active integrative element. The evidence for this assertion needs to be stated succinctly. Moreover, if this is the case I find it perplexing that it has been given a Tn number since it is clearly not a transposon. I appreciate that historically some integrative elements were assigned Tn numbers, but the important distinction between IE and Tn (site-specific recombinase verses comparatively random transposase) has been understood for some time. The practice of assigning Tn numbers to IEs promotes confusion and is hence unhelpful; it should not be continuing in 2021 - alternative naming conventions are being used for IEs. A complicating issue here is that at this time it is unclear what type of IE this one represents. It does not appear to be an ICE, but it could be an IME (integrase-like proteins have been found to serve as a relaxase in some conjugative plasmids) or it could be a form of a CIME. In any case, a name that is not misleading would be preferable.

(Response) In the revised manuscript, we explicitly stated that “This 7.1-kb region was confirmed to be active in generating its circular copy of top strand in *E. coli* and *V. alfacsenis* and inserting it into new target location in *E. coli* in this study“ (thus satisfying the definition of transposon in Roberts et al., 2008) at line 129-131.

While the term transposon/transposition has been clearly defined in several reviews and papers (ICE is classified as one form of transposons in Curcio and Derbyshire 2003), the term IE has not been explicitly defined historically. The definition of ICE/IME/CIME, based on their transfer activity, is also problematic in that the scientists cannot tell whether the new IE is CIME or not, if conjugation genes are not embedded in the IE.

In this revision, we conducted additional database surveys and found that Tn6945/SE-6945 and Tn6283/SE-6283 -like IEs clearly have 4 conserved genes (**new Fig** **2** and **new Fig 6**), and form a family of new mobile elements. They do not look like degraded ICE. In addition, the first step of their location change is presumably “copy-out” as elaborated in the revised manuscript, although their integration step is still vague (it can be copy-in (replication-dependent) or paste-in (replication-independent) in the classification by Curcio and Derbyshire 2003). Therefore, we coined the term SE for these IEs in the revised manuscript.

We think that frustration for the usage of transposon/transposition/integration/translocation for diverse readers will be solved if we explicitly state the rules of term usage in the introduction as stated above.

Line 121-124: This section describes insertion sites in E. coli, shown in Fig 1D, but the insertion sites from the Vibrio strains (shown immediately above them in the same figure) are ignored in the text. If they are not informative they should be removed; but surely they are. Is the information consistent between the organisms? I would have thought a consensus motif for attB target sites might be worth deducing/showing?

(Response) We added new information about insertion locations of SE-6945 like elements (**new Fig 2: line 200-225**) and SE-6283 like elements (**new Fig 6, line 398-423**). They indeed show target site preference.

Line 125: Indicative values should be provided for "very similar".

(Response) We modified the sentence as “As pSEA2 showed > 99.9 % nucleotide identity on the aligned region to pSEA1”: line 155.

Line 128: "previously" should be "previous".

(Response) We deleted this phrase in the revised manuscript: line 158.

Line 130-131: Without first meaningfully describing the similarity relationship between pSEA1 and pSEA2, it is difficult to know whether deducing a precursor based on the presence/absence of a site-specific integrative element is appropriate.

(Response) We removed the relevant sentence in the revised manuscript.

Section starting line 133: This section starts by outlining a plausible scenario for plasmid integration via a two-step process involving homologous recombination. However, analogous mobile element flanked plasmid co-integrate arrangements can also arise in a single step in the absence of homologous recombination. The possibility of this (and possibly other) scenarios needs to be noted here also so the rationale for the subsequent experiments is clear to a reader.

(Response) We think that a Tn3-like movement (Shapiro’s intermediate formation) is unlikely for SE for a couple of reasons. The formation of Shapiro’s intermediate requires one nicking at the top strand and one nicking at the bottom strand simultaneously. We actually show that strand exchange occurs only at the top stand (**Fig 4 DE**), thus, cointegrate formation through Shapiro’s intermediate is very difficult to happen. If cointegration formation happens, insertion of Tc Ap should occur as frequently as Ap insertion, which was also not the case for our observation.

To eliminate the frustration of readers, we added the following statement in line 256-261: “The Tn3-like cointegrate formation [15] might happen if SE-6945 could simultaneously introduce nick at one site on the top strand at attL (or attR) and one site on the bottom strand at attR (or attL), and DNA-protein crosslink (DPC) repair [38] could clean the linked tyrosine recombinase to produce a 3′-OH priming site. If SE-mediated cointegrate formation occurs, then Tc Ap-resistant transconjugant should emerge at a comparable frequency to Ap-resistant transconjugant, which was not the case in our observation.”

The subsequent two paragraphs, and Fig 2, which describe the data, are very difficult to understand in terms of what was done and the data resulting. It all needs to be described much more clearly. Plasmid integration is said to be based on transfer of both ampicillin and tetracycline, but then only tetracycline-resistant transconjugants are described, and only Tet + Ap is shown in Fig 2. Elsewhere in the paper, transfer frequency is provided "per donor" as is established practice. Frequencies indicated in Fig 2 should do likewise, rather than per donor x recipient. Moreover, I think it would be preferable for this data to be provided in a table rather than a graph, so it is clear what data was obtained for each mating. For example, the single dot for Tet+Ap transfer in the delta-recA strain does not really agree with what is stated in the text.

(Response) We changed the unit of Y axis of transfer frequency data (**new Fig 3**) to T/R. The reasons why we used log T/(D*R) is already mentioned above. We modified the description of “tetracycline resistance” to “Tc Ap resistance”. Data point for no transconjugant is indicated by open circle in the new **Fig 3**.

What is the explanation for the apparently reduced Ap transfer in the delta-recA strain? Could this be responsible for the failure to detect any Ap + Tet transfer (i.e., insufficient sensitivity)? If so, the data doesn't really provide clear support for the proposed two-step scenario.

(Response) We appreciate the reviewer’s comment. To address this, we added the following statement “Unexpectedly transfer frequency of Ap resistance was also reduced to the 1/17 level of JW0452rif in JW0452ΔrecArif (compare the second and the fourth groups from the left in Fig 3). Thus, the direct effect of recA knockout on pSEA2 integration could not be evaluated due to the reduced detection sensitivity of gene transfer. However, when LN52rif (which already carries one copy of SE-6945 and wild type recA) was used as the recipient, Tc Ap-resistant transconjugants were obtained at a 56-fold higher frequency (in mean) than that observed when using JW0452rif as the recipient (compare the first and fifth groups in Fig 3). This indicates that the presence of the 7.1 kb long homology stretches in both target DNA and pSEA2 DNA promotes pSEA2 integration in the recipient” in lines 247-256.

Section starting line 168 (to 191) and manuscript title: Copy-out-paste-in has been employed to denote a mechanism of transposition. The mechanism employed by this type of integrative element may indeed share some commonalities (which are certainly worth noting), but the enzymes involved and the biochemistry among other things are going to be different (e.g., the observed strand bias, as noted in the Discussion). I therefore feel that simply using the same Copy-out-paste-in term to describe this integration mechanism will again lead to confusion, leaving some readers with the impression that transposition and integration are the same when they are clearly not.

Additionally, mechanisms used by other integrative elements are not explained anywhere in the manuscript. Are they different? Does the data here preclude them?

(Response) All IEs discovered to date follow excision-(transfer)-integration route. We clearly stated this in the introduced section in the revised manuscript as “ICEs/IMEs so far studied in the laboratory seem to change their location via the ‘excision-integration’ mode only (line 69-71).

The failure to detect an empty attB site by PCR of itself does not represent compelling evidence that a copy of the element is retained in the donor replicon. Maybe the empty site is not re-joined and is hence lost from the population.

(Response) Actually, “not re-joined”, leaving one single strand bridge (figure-eight form), is the key feature of the first step of the original copy-out paste-in transposition of IS (Chandler 2015). The reviewer might have meant to say “Not re-joined” after two rounds of strand exchange. We think this is unlikely, because two rounds of strand exchange at the bottom and top strands generate the so-called ‘couple sequence’(heteroduplex) observed in the circular form of Tn916, and the sequencing of the spacer sequence between C and C’ in the cloned *attS* should detect two sequences, but we detected only one spacer sequence in *attS* in strain LN95.

In the revised manuscript, we used more words to explain our interpretation and chose to use **Path 1** (new Fig 4) in “copy-out route” instead of “copy-out paste-in transposition” since the integration step of SE is still vague. The updated **Fig 4A, Fig 4 legend,** and new descriptions added in lines 280-295 and lines 342-358 would help readers grasp all possible routes of transposition and understand our interpretations.

Was a positive control performed to show that an attB site could be detected for a different element that is known to generate them?

(Response) PCR detection of empty *attB* during IE excisions has been reported for representative ICE/IMEs. Those literature are cited in the introduction with the statement in lines 69-71 (reference 22-25) in the revised manuscript. We believe that it is not necessary for us to conduct the experimental analyses using those elements.

Indeed, could the attB PCR detect the empty site of CAIM 1831? Without additional evidence, it is premature to conclude that a copy-out-like mechanism is used.

(Response) We disagree with this reviewer’s comment. Since occupied *attB* (7.2 kb) was amplified using the same primers as used for empty *attB* detection (**new Fig 4 B)**, primers and PCR conditions are optimal. It is not necessary to test the genome of CAIM 1831. In our previous study (Nonaka et al 2018) on SE-6283, empty *attB* was detected, though at a very low frequency. The ratio of *attS* to empty *attB* was about 100:1 based on qPCR for SE-6283 in E. coli.

Line 215: Why is Tn6283 mentioned here? It doesn't seem to encode resistance so how is it relevant to these studies?

(Response) We here mentioned SE-6283 in 04Ya108 as another example of the occurrence of more than one SE in the genome. The occurrence of more than one copy in a genome may also be a feature of SE. Since there are only two examples of SE to date, SE-6283 is relevant to this section. Line 370-372.

Line 228: Although the results are mostly consistent with a correlation between Tn6945 copy number an Ap MIC, it is an overstatement to say they "clearly" show copy number affects resistance level; there is only a single strain with three copies, some strains with one or two copies exhibit low resistance, while another with one copy exhibits as much resistance as the most resistant isolates with two copies. This suggests that there are other factors at play so the data should be interpreted conservatively.

(Response) We appreciate the reviewer’s comment. When polishing the descriptions about the Southern hybridization results and new Table 1 (strain table), we noticed that the insertion location of SE also affects the MIC. Hence the paragraph title was changed as “The insertion copy number and location of SE-6945 affects β-lactam resistance levels of the host cell” in the revised manuscript” in lines 369-370. We also added the following descriptions in lines 388-391: “MIC of individual strain is shown in Table S1. When focused on a class of MIC 500, there was a trend that strains with SE-6945 insertion into insJ show a higher MIC than strains with insertion into yjjNt. Thus, the location of SEs can also affect phenotype variation, possibly due to the differences in the expression levels of their cargo genes.”

## Fig 4 legend: What do the different colours denote?

## (Response) The colors of the dots denote the copy number of SE. The phrase of “four groups shown in distinct colors” was added to the legend (new Fig 5. Line 394).

## Discussion line 256: The conviction in the two step mechanism is not justified by data presented. Other alternatives, and known mechanisms of other integrative elements should be elaborated.

## (Response) As we stated in response to reviewer 1’s comment, Tn3-like movement (Shapiro’s intermediate formation) is unlikely for SE, since the formation of Shapiro’s intermediate requires one nicking at the top strand and one nicking at the bottom strand simultaneously, as well as protein-DNA link repair for the SE to generate priming site. We actually found that nicking occurs only at the top stand for SE (**Fig 4 DE**).

## Furthermore, the presence of SE in the recipient still increases the pSEA2 integration frequency by 10-fold. For these reasons, homologous recombination is still the primal route of the pSEA2 integration into the chromosome.

We rephrased the sentence to be more conservative as follows “The current most likely mechanism of this phenomenon is the transposition of SE followed by homologous recombination” at line 446-447 in the revised manuscript.

Discussion line 275: The possibility that this element might be mobilisable (i.e., an IME) should be canvassed, particularly given the multiple genes of unknown function. However, all that might be needed is an oriT site. Has a candidate oriT sequence been identified in pSEA1/pSEA2 (likely near their relaxase gene), and is there a similar sequence in Tn6945?

(Response) The difficulty with pSEA1/pSEA2 is that they belong to MobH group, which is the least characterized plasmid/ICE group. The operonic structure of tra operon is not conserved among MobH group members.

Inverted repeat near oriT of Neisseria gonorrhoeae genomic island (GGI) encoding MobH family relaxase TraI, has been determined to be 5’-CAAAGGCCTGCATTTTTATGCAGGCCTTTG-3’ (Heilers et al 2019 NAR).

oriT of ICE-SXT contain Inverted repeat motif: 5’-ccaaaagccaaacggatagtggttttggcttttgg-3’ near oriT (Ceccarelli et al JB 2008), but it is not located immediately upstream of TraI relaxsase. We could not find a similar motif on pSEA1/pSEA2 and SEs based on similarity search. If CDS2 or CDS4 product was related to relaxase (HUH endonuclease), motif search in Pfam and InterproScan should show hits. But they did not. For these reasons, positive comments on the presence of *oriT* on SE would become a speculation based on speculation.

We understand that readers might show interest in whether SE possesses oriT or not. We added the following statement: “Another dissimilarity between SE and ICE/IME is that the former does not encode HUH endonuclease. There is still a possibility that SEs carry *oriT* recognized by a family of relaxases and are mobilized like CIME, though we could not identify *oriT*-like sequences in SE-6945 and SE-6283 by local similarity search alone” at line 494-497.

Line 573: The legend shown is presumably for S3 rather than S1. In any case, I doubt enough information is provided for a reader to understand the figure.

(Response) We corrected the legend names (current S1 Fig and S5 fig). We added more information for the Southern hybridization results as described above.

Line 581 and S2 legend: "...arch..." should be "...arc...". I could not find the significance of the "contig 1" described anywhere so it should be removed (from here and the figure). What is the meaning of the gene colours used in B?

(Response) We appreciate the reviewer’s comments. We added the sentence “The 14.2 kb *mphA*-*cat* gene region flanked by repeats in pSEA2 (S2 Fig A) was detected as a separate circular contig in the TJ249 genome assembly.” to the main text (Line 165-167) to describe the results clearly.

We also add the following statement in the legend ”Four SE core genes are indicated by four distinct colors. Red pentagons are the β-lactamase gene”. Line 888-889 in the revised manuscript.

Fig S2A: Are putative conjugation or replication initiation genes evident in the pSEA2 sequence? If so, it would be informative if these additional genes at least could be indicated on the figure.

## (Response) We added information on TraI relaxase/TraD coupling protein locations to the figure panel. Rep and oriV were difficult to predict (new S2 Fig).

## Fig S2B: pSEA1 should be included in the figure label. The legend provided is inadequate - what do the gene colours mean? The figure shows locations of the elements, not "insertion positions", and for both plasmids, not just pSEA1.

## (Response) We added legend for intA, intB, CDS2, CDS4, then corrected panel B title to be “Location of SEs in pSEA1 and pSEA2” (new S2 Fig).

6. PLOS authors have the option to publish the peer review history of their article ([what does this mean?](https://journals.plos.org/plosone/s/editorial-and-peer-review-process#loc-peer-review-history)). If published, this will include your full peer review and any attached files.


If you choose “no”, your identity will remain anonymous but your review may still be made public.


**Do you want your identity to be public for this peer review?** For information about this choice, including consent withdrawal, please see our [Privacy Policy](https://www.plos.org/privacy-policy).

Reviewer #1: No

Reviewer #2: No

[NOTE: If reviewer comments were submitted as an attachment file, they will be attached to this email and accessible via the submission site. Please log into your account, locate the manuscript record, and check for the action link "View Attachments". If this link does not appear, there are no attachment files.]

While revising your submission, please upload your figure files to the Preflight Analysis and Conversion Engine (PACE) digital diagnostic tool, <https://pacev2.apexcovantage.com/>. PACE helps ensure that figures meet PLOS requirements. To use PACE, you must first register as a user. Registration is free. Then, login and navigate to the UPLOAD tab, where you will find detailed instructions on how to use the tool. If you encounter any issues or have any questions when using PACE, please email PLOS at [figures@plos.org](mailto:figures@plos.org). Please note that Supporting Information files do not need this step.
