# Supplementary material for: Atypical integrative element with strand-biased circularization activity assists interspecies antimicrobial resistance gene transfer from Vibrio alfacsensis
Source: PLoS One. 2022 Aug 2;17(8):e0271627. doi: 10.1371/journal.pone.0271627 (PMC9345347; doi:10.1371/journal.pone.0271627)
Supplement: Attachment — Submitted filename: 2nd_comments_to_Reviewers.docx [file pone.0271627.s013.docx]

Responses to the Reviewer’s comment

PONE-D-21-18582

Reviewer #1: This revised manuscript includes interesting and important information about a new type of integrative element, and the scientific aspects have been improved by adding/clarifying experimental details. However, further revision is still needed to address some scientific points and better present the data and conclusions, as the manuscript is still hard to follow - information needs to be presented in a more logical order - and the text is still wordy and repetitive and could be condensed in places. I have tried to give some more detailed suggestions of how this could be done in the comments below.

(Reviewer #1’s Comments)

Nomenclature for the new integrative elements

(Response) In the revised manuscript, we have explicitly stated that the unclassifiable integrative elements, identified in this study, are a new class of mobile elements. Therefore, we have used an “SE-“ prefix (strand-biased circularizing integrative element) to refer to these IEs in the revised manuscript. We believe that using numbers to identify these DNA elements would make it easy to handle (refer to) them in existing and future mobile DNA databases. Therefore, we have used numbers to refer to the SEs in this manuscript.

1. NEW COMMENT: “SE” is not a particularly descriptive name. Including at least “I” for “integrative” should be be considered (i.e., “SIE”?).

(Response) We thank the reviewer for their comment. However, SIE may be confused with eukaryotic “SINE” transposons. Thus, we did not use it. When priorizing distinction among mobile DNA families, SE is rather apt. Putative integrative element Crypton in fungi does not even include I or E.

(Reviewer #1’s Comments)

2. Also, it might be better to introduce this term only after describing identification of the strand biased mechanism.

(Response) We have revised the manuscript based on this suggestion. In the Abstract, the word "Tn6283" has been used before the SE term definition (line 36). Similar to the Abstract, the word "Tn6283" has been used before the SE term definition at line 95 in the Introduction. As SE is defined in the Introduction, we see no issues with using the term "SE" in the Results.

(Reviewer #1’s Comments)

3. The Results sections could be reorganised to first describe the experiments that led to this conclusion (i.e., move lines 278-367 to after lines 168 and maybe combine lines 169-79 with it, ending with lines 129-131.

(Response) I am afraid that the section order suggested by Reviwer 1 is not really the best to explain the findings in this study. We attempted to formulate the text following Reviewer 1’s suggestion, but it resulted in restricted word usage in the figures (we could not use the terms “SE”, “attL”, “attR”, “attS” in the figure showing the PCR results) and induced repetition of the words ‘7.1 kb element’ in the Results section. Those were not really helpful.

Strand-biased circularization mechanisms were identified in our previsous study on Tn6283, and this has been alluded to in the revised manuscript (line 93). We then introduced the term “SE“ in the Introduction (line 95). Whether the 7.1 kb element is SE or not is relatively easy to deduce based on the results of genome sequencing. Therefore, introducing "SE-6945" at a relatively early location in the Results with belief reasoning and inclusion of the statement “(see below)” help reduce repetition of "the 7.1 kb element" in Figures and text and is rather apt.

(Reviewer #1’s Comments)

4. Also consider the best order for Results sections on lines 181-276 and 369-415- e.g., combining the sections on target preference for SE-6945 and SE-6383, or at least placing them together?

(Response) We agree with Reviwer 1 regarding this point. We combined the target site preference sections for SE-6945 and SE-6283 and made one new section "Target site preference of SE-6945 and SE-6283" (line 174).

(Reviewer #1’s Comments)

5. In the Abstract, the name SE-6945 is used on line 28, before the term SE is explained on lines 34-5. The explanation should be placed earlier, first stating something like “This 7.kb element has four coding sequences, which are related to those of an element previously referred to as Tn6283, two of which… PCR and sequencing revealed these elements generate a copy of only one strand…, so they are collectively termed SE (…) here.”

(Response) We modified the phrases and changed the order of sentences in the Abstract as suggested (lines 28 to 34).

(Reviewer #1’s Comments)

6. Also, the name SE-6283 is used on lines 80, before the term SE is explained on lines 93-5 – again suggest “called Tn6283”, but delete “(renamed…)” here and use Tn6283 for the rest of the paragraph, and “a related element” on line 91, then explain the term.

(Response) “SE-6283” in Introduction in the previsous mansucript was replaced with Tn6283 before line 95. Introductory sentences for the SE term are now “Based on the findings in the previous study and this study, we have termed the currently unclassifiable integrative element, Tn*6283*, and a related element as a new class of mobile elements: strand-biased circularizing integrative element (SE). Tn*6283* is hereafter referred to as SE-6283”.

(Reviewer #1’s Comments)

7. Another part of the response states “We prefer to use 4-digit numbers to keep this compatible with the existing Tn database, and in part considering the ease of handling them in future databases” but on line 205 the names “SE-VhaWXL538” is used, from the strain ID. Is the idea that this would be numbered at some point and if so, would the Tn registry be responsible for assigning the numbers? (also lines 502-3).

(Response) Yes, Tn registry assinged "9645". Tn registry assigns four digits to all DNA segments that satisfy the criteria used to define transposon (evidence of transposition) when requested. Bioinformatically screened SEs do not exhibit "evidence of transposition". Thus, the Tn registry would not assign numbers to them, but we need to refer to those putative transposons/SEs somehow in the text. Strain ID is a convenient and reasonablely clear solution for handling putative SEs in manuscript.

In the revised manucript we clearly mentioned the connection between name and the Tn registry at line 131: “Thus, the 7.1 kb element was labelled a transposon and assigned a four digits identifier, 6945, by the transposon registry “, and at line 506: “To refer to transposable individual SE members, we used digits that are compatible with the existing transposon database”.

(Reviewer #1’s Comments)

8. Introduction, lines 96-107, Use of transposition, integration insertion etc, L

The authors have explained why they prefer to use transposition as a more general term but I think that it would be better to incorporate these definitions with lines 53-72 and end the Introduction with the statement about identification of a new integrative element. Lines 104-7 (translocation) can simply be removed, as they are not relevant. I also think the use of the terms “integration” and “insertion” might be better reversed, to follow the traditional definition of integration, if SE are being called integrative elements. To me, SE-6945 integrates in the traditional sense, but the plasmid “insertion” is more complex, as it requires an additional recombination step.

(Response) We removed the terms “translocation”, and “insertion” and then introduced “cointegration” to refer to plasmid insertion into chromosome (as suggested by reviewer 2). “Integration” was used to refer to site-specific recombination, as suggested. However, we feel incorporation of these general definitions to an earlier paragraph addressing MGE classification (previous lines 53-72) would distract readers. Hence, we have not moved these lines (line 90-98).

(Reviewer #1’s Comments)

9. Results, lines 110-180　NEW COMMENT: The title of this section could be reconsidered, as it also discusses “Tn6283” and other points and the section needs to be reorganized and condensed. Results of sequencing 04Ya240 could be moved up and if the sequencing of TJ249 helped identify SE-6945 then this information should be before the descriptions of the proteins.

(response) The paragraph describing the sequencing of TJ249 has been moved up as suggested (line 115), and previous Fig 1 Panel C has also been changed to Panel A.

(Reviewer #1’s Comments)

10. The proposed gene product names are used on lines 127-8, but not explained until lines 132-4 – they should be explained first and then used.

(Response) We removed the explanations regarding product names because gene/product names were given to Tn6283/SE-6283 in the previous work.

(Reviewer #1’s Previous comments)

PREVIOUS COMMENT: 5. Lines 115-116 – the evidence for this is from the conjugation experiments?

(Response) We used the term “active” based on the evidence for (i) the circular form generation in the absence of pSEA2 in V. alfacsensis (new Fig 4, previous Fig 3) and plasmid-free E. coli (new S6 Fig) and (ii) new junction generation (attL, attR) in E. coli during conjugation experiments. We rephrased this sentence to explicitly state this fact as “This 7.1-kb region was confirmed to be active in generating its circular copy of top strand in E. coli and V. alfacsenis and inserting it into new target locations in E. coli in this study” in lines 129-131.

11. NEW COMMENT: As referred to above, I think that this information is in the wrong place. The point about the element being active and the top strand being involved should be after the description of the experiments that identified this (“to be active in generating its circular copy of top strand” also needs rewording).

(Response) We disagree with this comment. By providing the information about SE-6945 in the first, we can avoid repetitively describing “the 7.1 kb element” in figures and text. Adding “(see below)” would suffice since the readers can validate it after reading the paragraph about circularization detection.

Regarding rewording, we rephrased the sentence as follows: “As the 7.1-kb element was confirmed to generate a circular copy of one specific strand without leaving an empty site like SE-6283 in this study (see below)”. (line 132)

(Reviewer #1’s Comments)

12. Lines 164-7 are very unclear. The information about the mph(A) cat region is confusing. What kind of repeat? A repeated mobile element? Direct repeats/TSD? I couldn’t access the Figshare directly from the link in Ref 72/Table S2 (please check this) but I did manage to find the sequences. Contig names seem to be the other way round, i.e. contig2 is 14.2 kb. It looks like pSEA2 was transferred to E. coli and inserted, but a composite-transposon like structure was lost as a circular molecule? This all needs to be written so that it is much easier to follow and so that it does not get in the way of the main point about the two insertions in the chromosome.

(Response) The following sentence was deleted, as it was not relevant to the SEs (also mentioned by Reviewer 2).

“*The 14.2 kb mphA-cat gene region flanked by repeats in pSEA2 (contig 1 in S2 Fig A) was detected as a separate circular contig in the TJ249 genome assembly*” (previous lines 165-167).

(Reviewer #1’s Comments)

13. Line 122 – the gene name would be blaCARB-19, with bla in italics and CARB-19 as subscript, but the protein is not the same as CARB-19 in the NCBI reference gene catalog. As NCBI now assigns bla protein names/numbers it would be better to use their nomenclature, obtaining a name/number from NCBI if possible.

(Response) We communicated with the Pathogen-Detection group at NCBI, and they have assigned a new family name and a new allele name for this beta-lactamase gene. Now, *bla_GMA-1_* is used in the manuscript. We have added the following sentence at line 120: “One β-lactamase gene (60 % product identity to *bla_VHH-1_* in the CARD database) was located within a 7.1-kb repeat region found in both chromosome 1 and pSEA2 (S2 Fig B). This β-lactamase gene has been assigned a new family name, *bla_GMA_* (Gammaproteobacterial Mobile Class A β-lactamase), and allele name, *bla_GMA-1_,* in NCBI.”.

(Reviewer #1’s Comments)

PREVIOUS COMMENT 7. Lines 122-4 – “Tn6945” inserts itself, rather than its IR? It’s not clear whether it is being proposed that “Tn6945” has IR, like a typical e.g., Tn3 family transposon (and why are the repeats called C and C’?) which are internal to such Tn, or it has an att site that recombines with target att site to create hybrid sites, as for integrative elements. att site are mentioned in the Fig. 1 legend but are not discussed until later in the text, which is also a problem.

(Response). We understand the reviewer’s concern. We changed the phrase to “SE-6945 inserts itself ending with 5’-GTA-3 (Fig 1 A) along with an additional 6 bp next to the 5’ end of C at attL on the donor molecule into the target site” (line 177-178). C and C’ are from core site of site-specific recombination mediated by tyrosine recombinase (i.e. Lambda Int). The problem with this new integrative element is that we cannot deduce how much longer does the cis-element involved in recombination extend from motif C/C’. Therefore, we simply defined the term attL and attR as “The border regions between SE-6945 and its target locations” and then stated “The border regions between SE-6945 and its target locations were termed attL (intA-proximal side) and attR (intA-distal side) (Fig 2 A)” in the revised manuscript (line 169-170). We also elaborated to explain the feature around nicking sites at attL and attR at lines 170-176 and lines 210-216.

14. NEW COMMENT on lines 169-179 - see comments above on moving this section. Line 141 – please explain in the text why the names C and C’ are used. Also please consider whether just referring to border regions, rather than att, until the ends of the element have been properly defined would be better.

(Response) We have added a statement explaining why the terms C and C’ are used at line 152: “; the terms C and C’ were used because they were thought to be functionally equivalent to the core-type sites of phages [35].”.

Regarding attL/R, we think that the use of “border region” instead of attL/R would rather confuse readers (particularly in the figure). Therefore we did not follow this suggestion. Most Phage/ICE -related studies use the term attL/R without having a clear definition.

(Reviewer #1’s Comments)

4) Other scientific points

15. Line 61 – it is the attC site only that is folded.

(Response) We have deleted “a folded single-strand DNA” from the sentence and simply stated that “DNA insertion involving DNA replication can also be seen in the integration of integron gene cassettes into *attI*, and the phage CTX into *dif”* (line 62).

(Reviewer #1’s Comments)

16. Line 164 etc -is insJ associated with a known/named IS? If so, it would be better to use the IS name.

(Response) ‘*insJ*’ was replaced with ‘*insJ* of IS*3*’ (line 128).

(Reviewer #1’s Comments)

17. Line 239 – it would be helpful to note here that Ap resistance is on SE-6945, Tc resistance on the plasmid.

(Response) We have added the following line to the manuscript: “Note that Tc resistance is on pSEA2, while Ap resistance is on SE-6945”. (line 263)

(Reviewer #1’s Comments)

18. Line 255 – the “homology stretches” are actually identical?

(Response) Yes. The term “homology stretches” were deleted to avoid confusion and was replaced with “identical sequence” (line 280).

(Reviewer #1’s Comments)

19. Lines 287, 489 – do these descriptions apply to all IS? If not, “some IS” should be used.

(Response) We replaced “IS” with “some ISs” (line 313).

(Reviewer #1’s Comments)

20. Lines 288-9, 469– please reconsider descriptions relating to integron (upper case I not needed) gene cassettes and “empty donor sites”. The attC site belonging to a particular cassette moves with that cassette. Excision may create an empty attI if it was the only cassette, otherwise the downstream cassette moves up the array. Also, Ref. 40 on line 469 dates from before the single stranded mechanism was identified.

(Response) We modified the Discussion (not Results) section as follows: “In the replicative resolution model, strand exchange between two folded *attC* bottom strands and the following replication generates a gene cassette circle with *attC* and results in the removal of one gene cassette with *attC* from the gene cassette array on the donor molecule. This can cause formation of integron variants with reduced number of gene cassettes and even integrons with an empty *attI,* as observed in *in vivo* experiments [41].” (line464-469).

(Reviewer #1’s Comments)

21. 5) Table 1 This is useful but can be simplified and condensed. The Vibrio isolates need to be listed before the transconjugants and would also probably be better before the E. coli, with LN95 under 04Ya249. A heading “transconjugants” could be added, grouping these to reduce the size of the table, i.e., put LN# numbers for all isolates with identical characteristics on a single row (or even just list the number of transconjugants of each type, if LN numbers are not referred to elsewhere in the manuscript).

(Response) We reorganized Table 1 and reduced the size as per the suggestion. Now *Vibrio* isolates are listed first, followed by LN95, E. coli, and transconjugants. LN strains with identical characteristics were placed in a single row. (revised Table 1)

21.2 This would allow footnote c to be simplified e.g., note the selection used in the table itself and the text already states in several places that selections were done at 42 deg.

(Response) Table 1 contains information regarding Cm and Erm which were not used for transconjugants selection. Therefore, we cannot mix footnote information with Table 1 contents. Therefore, we have added the following lines: “Selection was done at 42 °C” (line 532) and removed three “42 °C”. However, antibiotic selection information remains unchanged.

The following sentences in previous manuscript have been deleted from footnote c. “*E. coli* strain LN52, which carries a single copy of SE-6945*,* is a JW0452 transconjugant obtained by ampicillin selection at 42 °C. TJ249 is also a JW0452 transconjugant obtained by erythromycin selection at 42 °C.”

(Reviewer #1’s Comments)

22. Footnote b is hard to understand.

(Response) We modified footnote b as follows: “*b.* Cointegration of plasmid pSEA2 with chromosome, independent of SE-6945 transposition. Location of pSEA2 in the transconjugant genome could not be defined based on Southern hybridization results.” (line 527).

(Reviewer #1’s Comments)

23. Please also align text in the first column on the left and at the top of each row, to make it easier to read.

(Response) The texts in the first column of Table 1 have been aligned to the left (revised Table 1).

(Reviewer #1’s Comments)

24. W0452deltarecA should be JW0452deltarecA?

(Response) Yes. We have replaced “W0452deltarecA” with “JW0452deltarecA” (new Table 1).

(Reviewer #1’s Comments)

25. Are all resistance genes are on the plasmid in each case? This should be made clear.

(Response) We have added the following sentence: “It is unclear whether the resistance genes on pSEA2 are still in the original locations for other transconjugants besides TJ249.” (line 525).

(Reviewer #1’s Comments)

26. Lines 531-3, 565-7 could also be incorporated into Table 1.

(Response) Lines 531-3 and lines 565-7 from the previous version of the manuscript have been deleted and we used more simple expression: “Ap for LN52” (line 533) and “Ery for TJ249” (line 534) in footnote c.

(Reviewer #1’s Comments)

27. 6) Figures Fig. 1A– the arrowheads might be better left off, as the reasons for proposing these nicking sites have not been introduced at this point in the text and showing them on a sequence is clearer /more informative. I would also suggest placing part C first to better follow the order in the text.

(Response) We have removed the arrowheads from previous Fig. 1 panel A (new panel B) and placed previous panel C fist in the new Fig.1, as suggested.

(Reviewer #1’s Comments)

28. Fig. 2 – see suggestions on reorganisation of text. As the two strains at the bottom of Part A don’t appear to have been used in Part B maybe these diagrams could be moved to Supplementary?

(Response) As mentioned in previous comments, reorganization would not enhance readers’ understanding. We chose to mention target site selection immediately after the sequencing results, as in the original manuscript. We think that those two diagrams are very useful to show that chromosomal target location of SE-6945 in genus Vibrio is limited. As *attB* in the chromosomes of strains WXL538 and 04Ya249 is occupied by an SE, we cannot define the ancestral *attB* sequences of those two strains. Hence, we used the unoccupied *attB* sequences of *V. harveyi* WXL538 and *V. alfacsensis* CAIM1831 as the ancestral *attB* sequences of strain WXL538 and 04Ya249 respectively in panel B. Therefore, SE target sites in the two strains of *V. harveyi* WXL538 and *V. alfacsensis* CAIM1831 still remain in main Fig 2 panel A.

(Reviewer #1’s Comments)

29. Legend - line 220 “coordinates”.

(Response) We have made the suggested modification (line 218)”.

(Reviewer #1’s Comments)

30. Line 221 “sequences were” “unpredictable” is the wrong word here – suggest “the bases in the putative crossover region could not be defined and are shown as X”.

(Response) We have rephrased the sentence as follows: “the bases on pSEA2 in the putative crossover region could not be defined and are shown as X” as suggested (line 221).

(Reviewer #1’s Comments)

31. “one site on the left side on the top strand” is unclear and needs rewording.

(Response) We have mentioned that “position is shown by black arrowhead” (Line 222) in the revised manuscript.

(Reviewer #1’s Comments)

32. Fig. 3 – line 270 – “no transconjugants were”.

(Response) We have replaced “transconjugant” with “transconjugants were” (line 297).

(Reviewer #1’s Comments)

33. Fig.4 - Legend – line 310 “Design of” can be deleted.

(Response) “Design of” has been deleted (line 335).

(Reviewer #1’s Comments)

34. Primer names are very long – can PCR reaction numbers be put in primer table?

(Response) We have shortened the primer name (line 338, Table 2).

(Reviewer #1’s Comments)

35. Line 215 – suggest “as shown as separate lines, with SE in orange”.

(Response) We have rephrased the sentence as follows: “Two strands of DNA are shown as separate lines, with SE in orange” (line 339).

(Reviewer #1’s Comments)

36. “IS3 route”, “ICE/IME route” “gene cassette route” shown on the figure are not referred to in the legend.

(Response) We have added “, referred to as ‘IS*3* (copy-out) route’ at line 344. ‘ICE/IME route’ and ‘gene cassette route’ have been added at line 349 and line 350, respectively.

(Reviewer #1’s Comments)

37. Reduce overlap between description of parts A and B.

(Response) We have deleted “Numbers in the primer set correspond to primer numbers in panel A” (line 351).

(Reviewer #1’s Comments)

38. Line 329 – “as arrowheads”.

(Response) We have made the suggested correction (line 353).

(Reviewer #1’s Comments)

39. Lines 372-8 – legends should generally not contain methods.

(Response) Lines 372-378 in the previous version of the manuscript were not parts of figure legends.

(Reviewer #1’s Comments)

40. Fig. 5 – mention Ap in the title and add “Ap” before MIC on vertical axis label.

(Response) We added “Ap” to the title (line 393) and the vertical axis label in Fig 5.

(Reviewer #1’s Comments)

41. Fig. 6 - line 420 – “and carry”

(Response) We used “and carry” in the revised manuscript (line 248).

(Reviewer #1’s Comments)

42. 7) Supplementary Fig. S1 legend, line 870 “and TJ249”

(Response) We used “and TJ249” (line 648).

(Reviewer #1’s Comments)

43. Fig. S2 legend, line 885 – “genes were”,

(Response) We used “genes were” in the revised manuscript (line 888).

(Reviewer #1’s Comments)

44. line 887 “PacBio assembly”

(Response) We have removed the information regarding contig 1 from the main text and from Fig S2 in the revised manuscript as it was not relevant to SEs (comment from reviewer 2). “PacBio assembly” has also been deleted.

(Reviewer #1’s Comments)

45. Fig. S3 legend – reword “part of the mobile DNA unit of the SE element” (also Fig. S4 lines 902-3, Fig. S7 line 930).

(Response) We rephrased the sentence as follows: “Sequences in red are incorporated into circular copy of SE.” (line 896, line 904, line 926).

(Reviewer #1’s Comments)

46. Fig. S6 legend – suggest “(B) Relative copy number of attS in *V. alfansensis* LN95 (no pSEA2)”

(Response) We have modified the figure title as suggested (line 936).

(Reviewer #1’s Comments)

47. Relative to what?

(Response) We used “relative copy number to *gyrB”* (line 938).

(Reviewer #1’s Comments)

48. 8) Minor/formatting/English

The Methods section could be simplified/condensed by restricting information to what was done, rather than too much description of why, especially if already explained in Results. Some minor rewording for accuracy/clarity is also needed to make the manuscript easier to read (some suggestions are included below).

(Response) We have simplified and condensed the text. We agree with almost all of Reviewer 1’s comments and have revised the manuscript according to the reviewer’s suggestion.

(Reviewer #1’s Comments)

PREVIOUS COMMENT: 14. Line 240 – V. cholerae is not always from sea food?

(Response) V. cholerae is indeed not always from sea foods as described in the cited reviews. These descriptions are correct. Thus we did not make changes to the revised manuscript.

49. NEW COMMENT: now line 430. I don’t understand this response. If these Vibrios are not always from sea food then the wording should be changed. *Vibrio* is one of the major bacterial genera found in marine sediments [26], and is among the most common microbiota of wild and farmed shrimp [27] and fish [28, 29]. Several of the genus *Vibrio* are pathogens of fishes reared in aquaculture [30], while other subsets of *Vibrio* species, including *V. cholerae*, *V. parahaemolyticus,* and *V. vulnificus*, which are ubiquitous in relatively low-salinity sea water, are seafood-borne human pathogens [30, 31].

(Response) We have modified the sentence as follows: “, which are ubiquitous in relatively low-salinity sea water, are human pathogens from seafood and/or water etc. [49, 50]” (line 427).

(Reviewer #1’s Comments)

PREVIOUS COMMENT: 36. Line 184 – meaning actually “chromosomal copy of Tn6945 in V. alfacsensis is functional”?

(Response) Because only one SE-6945 copy is present in LN95, detection of circular SE-6945 in LN95 indicates that the cis-elements (attL/attR) and strand exchange enzymes encoded in the chromosomal SE-6945 is indeed functional, while those of SE-6945 on pSEA2 is obviously functional as its transposition was observed in E. coli. We explicitly stated that “This suggests that the cis-elements (attL/attR) and strand exchange enzymes encoded by the chromosomal copy of SE-6945 are also functional in V. alfacsensis” in lines 304-306 in the revised manuscript.

50. NEW COMMENT: This was a comment on wording only. I think that what I previously suggested is clearer

(Response) We have stated that “the chromosomal copy of SE-6945 in V. alfacsensis is functional” as suggested (line 329).

(Reviewer #1’s Comments)

PREVIOUS COMMENT: 38. Line 199 – “absence of pSEA-2 from”

51. NEW COMMENT: Sorry, this should have referred to line 299, now line 515, and is simply a wording issue. If pSEA2 is absent then it is not “in” LN95, so “from” is clearer/more correct.

(Response) We have used “the absence of pSEA2 from LN95” in the revised manuscript (line 519).

(Reviewer #1’s Comments)

OTHER NEW COMMENTS:

52. Lines 37-8 – could delete “in addition to conjugative plasmids”

(Response) “in addition to conjugative plasmids” before “SEs are” have been deleted (line 39).

(Reviewer #1’s Comments)

53. Line 61 - “of an integron cassette” or “of integron gene cassettes” and “the phage CTX“

(Response) We used “integration of integron gene cassettes into *attI* [16] and the phage CTX into *dif*” as suggested (line 63).

(Reviewer #1’s Comments)

54. Line 33 – “The copy number and location”

(Response) We used “The copy number and location” in the revised manuscript (line 36).

(Reviewer #1’s Comments)

55. Line 64 – “a Y-transposase”, “an S transposase.

(Response) We have used “a Y-transposase” and “an S transposase”, as suggested (line 65).

(Reviewer #1’s Comments)

56. Line 67 – please give full names for these elements in addition to abbreviations.

(Response) We expanded abbreviated terms as follows: “IME (integrative mobilizable element) [18], MGI (mobilizable genomic island), CIME (*cis*-mobilizable element) [19], or MTn (mobilizable transposon) [20].” (line 68-70).

(Reviewer #1’s Comments)

57. Line 92 – “the E. coli chromosome”

(Response) We have used “the *E. coli* chromosome” as suggested (line 94).

(Reviewer #1’s Comments)

58. Line 99 – “as” before transposition should be omitted.

(Response) We have omitted “as” (line 102).

(Reviewer #1’s Comments)

59. Line 114 – “and transconjugants selected on erythromycin”

(Response) We have used “transconjugants were selected on erythromycin at 42 °C” as suggested (line 113-114).

(Reviewer #1’s Comments)

60. Line 120 – please give the accession no. for the CAIM 1831 sequence here or in Fig. S1.

(Response) The accession numbers for CAIM 1831 replicons have been added to revised Fig. S1.

(Reviewer #1’s Comments)

61. Line 121 – “plasmid” is not needed before pSEA2.

(Response) The term “plasmid” has been deleted (line 122).

(Reviewer #1’s Comments)

62. Line 122 – “a 7.1 kb repeat region”, not “the 7.1 kb repeat region”

(Response) We have used “a” (line 122).

(Reviewer #1’s Comments)

63. Line 133 – suggest “predicted” rather than “estimated”.

(Response) We have used “predicted” (line 144).

(Reviewer #1’s Comments)

64. Lines 159-60 – maybe “also carries two copies of SE-6945, in the same locations on the chromosome and plasmid as in 04Ya249”?

(Response) We have rephrased the sentence as follows: “also carries two copies of SE-6945, in the same locations on the chromosome and plasmid as in 04Ya249” (line 140-141).

(Reviewer #1’s Comments)

65. Line 186 – “transconjugants were screened on Tc alone, …”

(Response) We have used “transconjugants were screened on tetracycline (Tc) alone,” (line184).

(Reviewer #1’s Comments)

66. Line 195 - “other transconjugants”

(Response) We have used “other transconjugants” (line 193).

(Reviewer #1’s Comments)

67. Line 198 – delete “the”

(Response) We deleted “the” before “SE-6945 transposition” (line 196).

(Reviewer #1’s Comments)

68. Line 201 – “searched for”

(Response) We have added “for” (line 199).

(Reviewer #1’s Comments)

69. Line 214 - “integrons”.

(Response) We replaced “Integron” with “integrons” (line 212).

(Reviewer #1’s Comments)

70. Lines 234-5 – “altogether in a recA-null”

(Response) We replaced “altogether in the chromosome of a *recA*-null mutant” with “altogether in a recA-null mutant” (line 260).

(Reviewer #1’s Comments)

71. Lines 243 – this needs rewording - transconjugants are not transferred.

(Response) We want to emphasize the fact that transconjugants were obtained, albeit at a low frequency. Thus, the statement remains the same in the revised manuscript (line 266). We confirmed that these colonies received resistance genes and not spontaneous mutants by conducting single colony isolation and PCR.

(Reviewer #1’s Comments)

72. Line 246 – “in any of the four”

(Response) We have used “in any of the four” (line 272).

(Reviewer #1’s Comments)

73. Line 248 – suggest “the transfer frequency of Ap resistance to JW0452deltarecArif was about 17 fold lower than transfer to JW0452rif”. Was this for the means?

(Response) Yes. These are for mean values. We rephrased the sentence as follows: “the mean transfer frequency of Ap resistance to JW0452Δ*recA*rif was about 17-fold lower than transfer to JW0452rif” (line 274).

(Reviewer #1’s Comments)

74. Line 253 - “in mean” needs rewording.

(Response) We rephrased the sentence as follows: “However, the mean transfer frequency of Tc Ap resistance to LN52rif (which already carries one copy of SE-6945 and wild type *recA*) was about 56-fold higher than transfer to JW0452rif.” (line 277).

(Reviewer #1’s Comments)

75. Line 256 – “integration into the recipient chromosome”

(Response) We rephrased the sentence as follows: “promotes cointegration of pSEA2 and the recipient chromosome” in the revised manuscript (line 281).

(Reviewer #1’s Comments)

76. Line 281 – fix “E. col”

(Response) Corrected. (line 307).

(Reviewer #1’s Comments)

77. Lines 282-3 – could this be expressed the other way round? i.e., tyrosine recombinase typically produce staggered cuts at att sites?

(Response) Staggered cuts indicate occurrence of a double-strand break, and are misleading (the product is different from the staggered cuts made by restriction enzyme). Therefore, we did not change the phrase (line 308-309).

(Reviewer #1’s Comments)

78. Lines 288, 291, 360 etc – “single stranded”, “double stranded”

(Response) We corrected “single-strand” and “double-strand” to “single stranded” (lines 311, 314, 316, 317, 342, 344, 349, 462, 463, 477, 490) and “double stranded” (lines 309, 343, 346, 348, 384) respectively, through the whole text.

(Reviewer #1’s Comments)

79. Line 297 – “anneals inside the integrative element and the other outside it”

(Response) We modified the sentence as suggested (line 323).

(Reviewer #1’s Comments)

80. Line 301 – “primer sets”.

(Response) The primer set name was 1-4, but not 1, 2, 3, or 4. Therefore, to avoid confusion, we added ‘ ’ and used ‘1-4’ instead of 1-4 (line 326).

(Reviewer #1’s Comments)

81. Line 308 – “long PCR conditions were used”

(Response) We used “were used” (line 332).

(Reviewer #1’s Comments)

82. Line 372 – “their genome”

(Response) We have used “their genomes” (line 396).

(Reviewer #1’s Comments)

83. Lines 379-80, 387- need rewording - only Ap MICs were obtained.

(Response) We used “Ap” instead of “antibiotic” (Line 403, line 411, new Fig 5).

(Reviewer #1’s Comments)

84. Line 388 – “MICs against individual strains are”. Not clear what “when focused on a class of MIC 500” means.

(Response) We have added the phrase “against individual strains” (line 405). The line “when focused on a class of MIC 500” has been deleted. (line 412).

(Reviewer #1’s Comments)

85. Line 405 – “screened for”, or “identified”

(Response) We have used “screened for” (line 231).

(Reviewer #1’s Comments)

86. Line 406 – “and related SEs”

(Response) We have omitted “its” as suggested (line 232).

(Reviewer #1’s Comments)

87. Line 431 – “linking” not really the right word here, “aiding movement between”?

(Response) We have used “aiding movement of” instead of “linking” as suggested (line 428).

(Reviewer #1’s Comments)

88. Line 462 - “transposon mechanisms”?

(Response) Here, we have used “movements” in the revised manuscript (as suggested by reviewer 2) (line 459).

(Reviewer #1’s Comments)

89. Line 469 – needs rewording

(Response) Please refer to our response to comment 20.

(Reviewer #1’s Comments)

90. Lines 288-9, 469– please reconsider descriptions relating to integron (upper case I not needed) gene cassettes and “empty donor sites”. The attC site belonging to a particular cassette moves with that cassette. Excision may create an empty attI if it was the only cassette, otherwise the downstream cassette moves up the array. Also, Ref. 40 on line 469 dates from before the single stranded mechanism was identified.

(Response) Please see our response to comment number 20.

(Reviewer #1’s Comments)

91. Line 473 - “unjoined”?

(Response) We used “nicked” because this word was suggested by the Language editor at Editage (line 487).

(Reviewer #1’s Comments)

92. Line 475 – “a process equivalent to”

(Response) We have rephrased the sentence as suggested (line 475).

(Reviewer #1’s Comments)

93. Line 496 – “relaxases”,

(Response) We have replaced “relaxase” with “relaxases” (line 497).

(Reviewer #1’s Comments)

94. “and oriT in SE-6945 or SE-6282”

(Response) We have replaced “SE-6945 and SE-6283” with “in SE-6945 or SE-6283” (line 498).

(Reviewer #1’s Comments)

95. Lines 567-8 would be better incorporated into lines 563-5 i.e. add “and incubated at 42deg C to supress growth of Vibrio strains” to the end of the sentence. See also comments above on incorporating information into Table 1.

(Response) We have moved up Lines 567-8: “and incubated at 42°C to suppress growth of Vibrio strains” (line 567).

The following sentence have been deleted: “The transconjugant strain TJ249 was obtained by the first mating experiment of strain 04Ya249 with *E. coli* strain JW0452 as the donor and recipient, respectively, followed by Ery selection at 42 °C”.

(Reviewer #1’s Comments)

96. References – species names should be in italics.

(Response) We have looked at the reference sections, carefully and confirmed that all species names are in italic (Lines 663-869).

Reviewer #2: The revision of the manuscript has clarified many aspects of the original version. I don't think it will be productive to argue the utility of general verses specific use of the words transposon/transposition. More importantly, I feel that the use of "SE" in place of "Tn" is a satisfactory solution that clearly implies distinctions between these particular integrative elements from others, and Tn-named elements.

Further suggestions:

(Reviewer #2’s Comments)

1. Line 25: "multidrug resistance conjugative plasmid" is probably better written as "conjugative multidrug resistance plasmid".

(Response) We have used "multidrug resistance conjugative plasmid" as suggested (line 27).

(Reviewer #2’s Comments)

2. Line 31: "of its one specific" can just be "of one specific".

(Response) We have used "of one specific" (line 33).

(Reviewer #2’s Comments)

3. Line 33: "Copy numbers and location" should be "The copy number and location".

(Response) We added “The” before “copy number (line 36).

(Reviewer #2’s Comments)

4. Line 48: Sentence should be: Conjugative plasmids and integrative and conjugative elements (ICE) [6] are DNA units that can move from one cell to another through conjugation machinery that they encode.

(Response) We have added “that they encode” as suggested (line 51).

(Reviewer #2’s Comments)

5. Line 100: Just a suggestion. Replicon fusion between plasmids and/or chromosome could be termed "cointegration", leaving "integration" to be used for site-specific recombination, since "insertion" is commonly used for events not involving site-specific recombination.

(Response) We appreciate the reviewer’s suggestion. We introduced the term “cointegration” to refer to plasmid-chromosome cointegration, and used “integration” for site-specific integration (also suggested by Reviewer 1). We did not declare usage for either “insertion” or ” translocation” in the revised manuscript. The terms definition paragraph (line 99) is now: “For clarity, terms *transposition*, *cointegration*, and integration are used here based on the following rules. We call the movement of a DNA segment from one location to the other location in one or two genomes, mediated by any DNA strand exchange enzymes, transposition. The physical incorporation of a plasmid into a chromosome is called cointegration regardless of the molecular mechanisms and genomic locations involved. The physical incorporation of a DNA molecule into a specific site in another molecule mediated by a site-specific recombinase (so-called integrase) are called integration” (line 99-106).

(Reviewer #2’s Comments)

6. Line 103: I appreciate the authors explaining why they haven't used the term "translocation". However, since it is not used the explanation isn't needed in the paper - it might appear perplexing to a reader.

(Response) We have deleted the sentence as suggested (see above).

(Reviewer #2’s Comments)

7. Line 114: "and the transconjugant was first selected" should be "and transconjugants were selected".

(Response) We have modified the sentence as suggested (line 113).

(Reviewer #2’s Comments)

8. Line 166 and Fig S2: The significance of contig 1 is not clear to me. How does it indicate movement of SE-6945?

(Response) All statements related to “contig 1” have been deleted from the revised manuscript and revised Fig. S2, as it is not relevant to SEs.

(Reviewer #2’s Comments)

9. Line 186: "transconjugant was" should be "transconjugants were".

(Response) We have used “transconjugants were” (line 184).

(Reviewer #2’s Comments)

10. Line 190: " generated intA-containing fragments originating from" should be "possessed intA-containing fragments located at".

(Response) We have used “possessed” (line 188).

(Reviewer #2’s Comments)

11. Line 197: It is more correct to say that pSEA2 integration relies on SE-6945 transposition, since generally transposition of SE-6945 will NOT result in plasmid integration (it will do so only rarely).

(Response) We added the lines ‘pSEA2 integration relies on SE-6945 transposition’ (line 195).

‘Since generally transposition of SE-6945 will NOT result in plasmid integration’ was not added, as this additional phrase might be rather distracting.

(Reviewer #2’s Comments)

12. Line 212 and elsewhere: "Sequence logos" should be "Sequence logo".

(Response) We replaced “Sequence logos” with "Sequence logo" (line 223).

(Reviewer #2’s Comments)

13. Line 257: "The Tn3-like" should be "Tn3-like".

(Response) We corrected "The Tn3-like" to "Tn3-like" (line 283).

(Reviewer #2’s Comments)

14. Line 347 and 353: (Fig 4E) should be (Fig 4D).

(Response) We have changed (Fig 4E) to (new Fig 5D) in line 377 and line 379.

(Reviewer #2’s Comments)

15. Line 371: "in genomes" should be "in their genomes".

(Response) The manuscript was revised as per the reviewer’s suggestion (line 395).

(Reviewer #2’s Comments)

16. Line 372: Why/how would/could SE-6283 be expected to influence beta lactam resistance? Seems like a complete red herring to me.

(Response) We used SE-6283 here as an example of an SE having two target sites in a genome, to emphasize that SE copy number in a genome can increase. In this study, we used SE-6945 carrying *bla* to illustrate that copy number of SEs in a genome can affect any phenotype. However, we felt that the phrase, “Furthermore, 04Ya108 also carries two copies of SE-6283” was not necessary to express our message. We have deleted this sentence in the revised manuscript (line 396).

(Reviewer #2’s Comments)

17. Line 388-391 paragraph: Could just be: The MIC of individual strains is shown in Table S1. There was a trend for strains with SE-6945 insertion into insJ to show a higher MIC than strains with insertion into yjjNt. Thus, the location of SEs can result in phenotype variation, possibly due to the differences in the expression levels of their cargo genes.

(Response) We have modified the sentence as suggested (line 412).

(Reviewer #2’s Comments)

18. Line 414: As written, the sentence implies the opposite of what is meant. I suggest change to "and, like attB of SE-6945 did not possess an inverted repeat structure."

(Response) We appreciate Reviewer 2’s suggestion. We have added “and, like attB of SE-6945 did not possess an inverted repeat structure” to the revised manuscript (line 240).

(Reviewer #2’s Comments)

19. Line 418: Isn't it five genomic locations shown in Fig 6A?

(Response) We appreciate the Reviewer 2’s comment. We have changed “six” to “five” (line 245).

(Reviewer #2’s Comments)

20. Line 462: "movements" instead of "moves"

(Response) We have changed “moves” to “movements” (line 459).

(Reviewer #2’s Comments)

21. Line 471: "possibly the CDS2" rather than "i.e., CDS2".

(Response) We have changed "i.e., CDS2" to “possibly the CDS2” (line 471).

(Reviewer #2’s Comments)

22. Line 473: " transposases" instead of "transposase".

(Response) We have replaced "transposase" with "transposases" (line 473).

(Reviewer #2’s Comments)

23. Line 497: It might be worth mentioning here that a tyrosine recombinase-like protein has been shown to function as a conjugative relaxase, particularly since these elements contain two such proteins. See reference <https://doi.org/10.1111/mmi.13270>.

(Response) We appreciate this useful information and suggestion by Reviewer 2. We added the following lines about the possible function of IntB as relaxase to the Discussion: “It may also be possible that IntB, a large tyrosine recombinase, functions as a relaxase, since a conjugative plasmid from *Clostridium* has been shown to use a tyrosine recombinase homolog as a conjugative relaxase.” (line 498-501). The relevant paper has been cited in the revised manuscript.
