# Supplementary material for: Atypical integrative element with strand-biased circularization activity assists interspecies antimicrobial resistance gene transfer from Vibrio alfacsensis
Source: PLoS One. 2022 Aug 2;17(8):e0271627. doi: 10.1371/journal.pone.0271627 (PMC9345347; doi:10.1371/journal.pone.0271627)
Supplement: Attachment — Submitted filename: R3_responce_letter.docx [file pone.0271627.s014.docx]

Responses to the Reviewer’s comment

PONE-D-21-18582

Reviewer #1: This revised manuscript is improved, and I have not commented further on the organisation, as the authors have provided reasons why they do not want to change this. The manuscript is essentially now acceptable for publication, but as PLoS One does not copyedit accepted manuscripts, addressing the few small points below, which include suggestions to further improve English/wording, would enhance clarity/readability.

(Reviewer #1’s Comments)

1) ORIGINAL COMMENT: 71. Lines 243 – this needs rewording - transconjugants are not transferred.

(Response) We want to emphasize the fact that transconjugants were obtained, albeit at a low frequency. Thus, the statement remains the same in the revised manuscript (line 266). We confirmed that these colonies received resistance genes and not spontaneous mutants by conducting single colony isolation and PCR.

NEW COMMENT: Sorry if this was not clear – it was simply a comment on the wording – “the transfer of Tc Ap-resistant transconjugants” does not make sense - transconjugants are not transferred, they are the result of transfer. It could be changed to e.g., “transfer of both Ap and Tc resistance”.

(Response)

We have used as “transfer of both Ap and Tc resistance” as suggested by reviewer 1 (line 268).

(Reviewer #1’s Comments)

2) ORIGINAL COMMENT 77. Lines 282-3 – could this be expressed the other way round? i.e., tyrosine recombinase typically produce staggered cuts at att sites?

(Response) Staggered cuts indicate occurrence of a double-strand break, and are misleading

(the product is different from the staggered cuts made by restriction enzyme). Therefore, we did not change the phrase (line 308-309).

NEW COMMENT: Lines 308-9 - “this assertion is based on the notion that tyrosine recombinases have a nature to not produce blunt ends at att sites on double stranded DNA during strand exchange”. This wording is quite convoluted – would e.g. “recombinases generally do not produce blunt ends” work?

(Response) We have used “recombinases generally do not produce blunt ends” as suggested by reviewer 1 (line 307).

3) Minor wording suggestions

(Reviewer #1’s Comments)

Lines 34-5 – “of integron gene cassettes”

(Response) We have changed “of the integron gene cassettes” to “of integron gene cassettes” as suggested (line 34-35).

(Reviewer #1’s Comments)

Lines 76-7, 83-4 – abbreviation AMR could be explained on lines 76-7.

(Response) We have introduced “antimicrobial resistance (AMR) genes” at lines 76-77, then used “AMR genes” at lines 83-84 as suggested by reviewer 1.

Line 89 – “elements”

(Response) We have changed "interspecies gene transfer assisted by Tn*6283*-like element is common" to “interspecies gene transfer assisted by Tn*6283*-like elements is common” as suggested (line 88-89).

Line 96 – suggest delete “the currently unclassifiable integrative element”

(Response) We deleted this phrase.

Line 99 – “movement”

(Response) We have changed “several types of intracellular DNA movements” to "several types of intracellular DNA movement”, as suggested (line 98).

Line 100 – “the terms”

(Response) We have changed “terms” to “the terms” (line 99).

Line 106 – “is called”

(Response) We have changed “are called” to “is called” (line 105).

Lines 121-3 – “VHH-1”, “GMA” and “GMA-1” should not be in italics.

(Response) We corrected (line 120, line 122 and line 123).

Line 130 – “insJ”

(Response) We have changed “*InsJ*” to “*insJ*” (line 129).

Lines 132-5 – need rewording for clarity, delete “member of”

(Response) “member of “ has been deleted.

Line 136 – “in the aligned region”

(Response) We have changed “of the aligned region” to “in the aligned region” as suggested (line 135).

Lines 151-3 suggest “SE-6283 and SE-6945 carry imperfect 19 bp inverted repeat motifs at their ends, (Fig 1 B), termed C and C’ based apparent on functional equivalence to the core-type sites of phages” (assuming “Fig. 1 fB is not correct here?).

(Response) We rephrased the sentence according to the reviewer’s suggestion. However, reference to “Fig. 1B remains because it actually shows the sequences of C and C’ below genetic map (line 150-152).

Line 169 – “the two E. coli chromosomal locations and the two locations”

(Response) We have changed “two E. coli chromosomal locations” to “the two E. coli chromosomal locations” as suggested (line 168).

Line 182 – “a specific location in the”

(Response) We have changed “a specific location of *E. coli* chromosome” to “a specific location in the *E. coli* chromosome” as suggested (line 181).

Line 185 – “The SE-6945 integration”

(Response) We have changed “SE-6945 integration pattern” to “The SE-6945 integration pattern” as suggested (line 184).

Line 187, 194 – “selection with Ap alone”

(Response) We have changed “Ap alone selection” to “selection with Ap alone” (line 186 and line 193).

Line 188 – “transconjuants, 12 had integrase…”

(Response) We used “transconjuants, 12 strains had *intA…*”, because integrase is protein name and gene name *intA* is rather appropriate here (line 187).

Line 199 – “the NCBI”

(Response) We added “the” before “NCBI” (line 198).

Line 201- “contains”

(Response) We used “contains” instead of “contained” (line 200).

Line 203 – “provisionally termed”

(Response) We changed “tentatively termed” to “provisionally” as suggested (line 202).

Lines 209-10 need rewording

(before update) Sequences of unoccupied target sites (*attB*) in *E. coli*, *V. alfacsensis*, and *V. harveyi* are shown in Fig 2 B.

(Response) We have removed “unoccupied” from the original sentence and the sentence is now simply “Sequences of target sites (*attB*) in *E. coli*, *V. alfacsensis*, and *V. harveyi* are shown in Fig 2 B (line 208-209).”

Line 211- “is T rich”, “structures”

(Response) We changed “was” to “is”, and “structure” to “structures”, respectively. The updated sentences are the followings: “The central 6 bp putative crossover region in *attB* is T rich. However, other motifs, such as imperfect inverted repeat structures observed in *attC/attI* of integrons (IntI target), or *dif* of CTX phage integration site (XerC/XerD target), could not be detected in *attB, attL* (S3 Fig), or *attR* (S4 Fig) for SE-6945 in the current data set” (line 209-213).

Line 240 “are G”

(Response) We used “are G” instead of “were G” (line 239).

Lines 241-2 - “suggest that, like ICEs, SEs target a few selected locations”.

(Response) We rephrased according to the reviewer’s suggestion (line 240-241).

Line 284 – “nicks”

(Response) We changed “nick” to “nicks” (line 283).

Line 291 – “mechanism, transposition followed by homologous recombination, rather than through

(Response) We changed “mechanism; transposition followed by homologous recombination than” to “mechanism, transposition followed by homologous recombination, rather than” as suggested by reviewer 1 (line 290).

Line 306 – “upon circularization”

(Response) We changed “upon its circularization” to “upon circularization” as suggested (line 305).

Line 314 – “or the generation of circular integron gene cassettes”, why “donor sites”

(Response) We changed “or the single stranded circle generation of Integron gene cassette by *attC* × *attC* recombination (gene cassette route), both of which generate empty donor sites” to “or the generation of cirucular integron gene cassettes by *attC* × *attC* recombination (gene cassette route)” (line 312-314).

We used “empty donor sites” to emphasize the common feature of ICE excision and gene cassette generation. The reviewer 1 might have confused with the definition of “donor site” on gene cassettes. We have removed “generation of empty donor site” from the sentence to simplify the sentence, as it will not affect our main message.

Line 332 - “when long”

(Response) We changed “when the long PCR conditions “to” “when long PCR conditions” (line 330).

Lines 372-3 – “at the C end”

(Response) We have changed “nicking occurs 6 bp upstream at the 5’ of C end and 3’ of C end” to “nicking occurs 6 bp upstream at the C end and the C′ end and/or 6 bp upstream at the C′ end and the C end”, as suggested.

(Response 2) We have changed nicking occurs 6 bp upstream of the 5′ end of C side and 3′ end of C′ side and/or 6 bp upstream of the 5′ end of C′ side and 3′ end of C side to “nicking occurs 6 bp upstream at the C end and the C′ end and/or 6 bp upstream at the C′ end and the C end”, as suggested (line 369-370).

Line 384 – “nicking of one strand of”

(Response) We have changed “Nicking at one strand on the double stranded DNA” to “Nicking of one strand of the double stranded DNA” as suggested (line 381).

Line 400 “The location”

(Response) We have changed “Location of SE-6945” to “The location of SE-6945” (line 397).

Line 412 – “of strains are”

(Response) “The MIC of individual strain is shown in Table 1” to “The MICs of strains are shown in Table S1.” (line409).

Line 413 – “integrated”

(Response) We have changed “SE-6945 integration into *insJ* “ to” to “SE-6945 integrated into *insJ*” (line 409-410).

Line 484-5 – “in transposition”

(Response) We have changed “SE-6283 transposition from” to “SE-6283 in transposition from”, as suggested (line 481-482).

Line 459 “Among the types of transposon movement”

(Response) We have changed “Among the transposon movements” to “Among the types of transposon movement” (line 456).

Line 486-7 – “with fewer gene cassettes”

(Response) We have changed “with reduced number of gene cassettes” to “with fewer gene cassettes” as suggested (line468-9).

Line 469 – “barely accompanies” not clear

(Response) We have changed “SE circle formation barely accompanies the generation of empty *attB*” to “SE circle formation rarely leaves the empty *attB*.”

Line 472 “of gene cassettes”

(Response) We have changed “of the gene cassette” to “of gene cassettes” (line 468-469).

Line 481 – “was repeatedly”?

(Response) We have changed “is repeatedly” to “was repeatedly” (line 477).

Lie 482 - delete “step”

(Response) We have deleted “step”.

Line 496 - “an HUH”

(Response) We have changed “HUH endonuclease” to “an HUH endonuclease” (line 493).

Line 505 - “numbers” rather than “digits”?

(Response) We have changed “digits” to “numbers” as suggested (line 503).

Line 514 – replace “strain 04Ya108” with “it”

(Response) We updated the sentence as follows:

(before update) Strain 04Ya108 was previously identified as *Vibrio ponticus* based on 16S rRNA gene sequence similarity. However, determination of the complete sequence in this study revealed that strain 04Ya108 shows >96% average nucleotide identity to *V. alfacsensis* strain CAIM 1831 (DSM 24595) S1 Fig B [67].

(after update) Strain 04Ya108 was previously identified as *Vibrio ponticus* based on 16S rRNA gene sequence similarity. However, determination of the complete sequence in this study revealed that it shows >96% average nucleotide identity to *V. alfacsensis* strain CAIM 1831 (DSM 24595) S1 Fig B [67] (line 509-512).

Line 516 “re-classified”

(Response) We have changed “newly classified” to “re-classified” (line 513).

Line 530 – “The 19 transconjugants listed ()”

(Response) We have changed “19 transconjugants listed” to “The 19 transconjugants listed” (line 527).

Lines 616-7 – “Ap concentrations”

(Response) We have changed “antibiotics concentrations” to “Ap concentrations” (line 613-614).

Line 632 – “searched for”

(Response) We have changed “searched“ to “searched for” (line 629).

Reviewer #2: The authors have satisfactorily addressed all my previous comments. A few minor corrections are listed below.

Minor corrections:

Line 27: "multidrug resistance conjugative plasmid" should have been changed to "conjugative multidrug resistance plasmid".

(Response) We have changed "multidrug resistance conjugative plasmid" to "conjugative multidrug resistance plasmid" (line 27).

Line 106: "are called" should be "is called".

(Response) Corrected (line 102) (reviewer 1 also pointed out).

Fig. 1C: The location of the conserved R at the top of Box I appears to be misaligned by two residue positions.

(Response) Thank you for pointing out this. Indeed, "R” was misaligned in the updated figure. We have uploaded corrected Fig F1.

After line 180: It would be helpful for readers for S5 Fig B was referred to specifically at the appropriate place.

(Response) We referred to S5Fig B in the following sentence (line 318-322). “we created a pSEA2-free 04Ya249 derivative strain LN95 carrying only a single copy of SE-6945 in the chromosome (S5 Fig B) and analyzed the production of its circular molecule in this strain and the sequence of strand exchange products.”

Line 196: "but not" should be "rather than".

(Response) We replaced “but not” with "rather than" (line 195).

Line 209: "nucleotide" should be "nucleotides".

(Response) We have changed "nucleotide" to "nucleotides" (line 208).

Line 210: "Sequence" should be "sequence".

(Response) We have changed “Sequence logo” to “sequence logo” (line 209).

Line 222: "trshown" should presumably just be "shown".

(Response) We corrected (line 221).

Line 249: "Sequence logos" should be "sequence logo".

(Response) We corrected (line 248).

Line 287: "transconjugant" should be "transconjugants".

(Response) Corrected (line 286).

Line 348: "circular a" should be "a circular".

(Response) Corrected (line 346).

Line 412: "strain" should be "strains".

(Response) Corrected (line 410) (reviewer 1 also pointed out).

Line 428: "water etc" should just be "water".

(Response) Corrected (line 425).

Line 496: "carry oriT" should be "carry an oriT".

(Response) Corrected (line 493-434).

Line 498: "identify oriT" should be "identify an oriT".

(Response) Corrected (line 495).

Line 502: "MDR conjugative plasmids" should be "conjugative MDR plasmids".

(Response) Corrected (line 499).

S5 Fig B legend. This legend is inadequate. It should at least say it is a Southern blot and give some insight into the probe used - can refer to S5 Fig A legend where it is shown (but not described). Likewise for the S5 Fig C legend.

(Response) We have increased information of S5 Fig B C legend, and referred to panel A as follows (line 911-918).

(B) Southern blots of the pSEA2-free *Vibrio* strain LN95 and the parental strain 04Ya249. Genomic DNA was double digested with *Nde*I and *Hin*dIII (the left two lanes) or *Nde*I and *Sph*I (the right two lanes). The probe used was 5’ end of *intA*. Four unique bands originate from fragments shown in the first two rows (04Ya249 pSEA2, 04Ya249 chr1) in panel A. (C) Southern blots of 19 *E. coli* transconjugants obtained from 19 independent mating assays. Upper panel shows digestion with *Nde*I and *Sph*I. Lower panel shows digestion with *Nde*I and *Hin*dIII. The color of the strain name indicates the pattern of SE/pSEA2 insertion deduced from restriction maps in panel A
